# Understanding discrepancies in noncovalent interaction energies from wavefunction theories for large molecules

**DOI:** 10.1038/s41467-025-64104-8

**Published:** 2025-10-14

**Authors:** Tobias Schäfer, Andreas Irmler, Alejandro Gallo, Andreas Grüneis

**Affiliations:** Institute for Theoretical Physics, TU Wien, Wiedner Hauptstraße 8–10/136, Vienna, Austria

**Keywords:** Quantum chemistry, Method development

## Abstract

Are the currently used reference methods to approximately solve the many-electron Schrödinger equation accurate enough? Here, we investigate recently reported discrepancies of noncovalent interaction energies for large molecules predicted by two of the most widely-trusted many-electron theories: diffusion quantum Monte Carlo and coupled-cluster theory. We are able to unequivocally pin down the main source of the puzzling discrepancies and present modifications to widely-used coupled-cluster methods needed for more accurate noncovalent interaction energies of large molecules on the hundred-atom scale. This is of critical impact for a wide range of applications which rely on highly-accurate interaction energies between large and polarizable molecules.

## Abstract (web summary)

High-level electronic structure methods present discrepancies for noncovalent interactions in large molecules. Here, the authors investigate these discrepancies and propose a modified coupled-cluster method for improved accuracy in large-scale molecular predictions.

## Introduction

The calculation of electronic transition energies for a single hydrogen atom in 1926 marks the beginning of an incredible successful era of quantum mechanics^1,2^. Shortly after this breakthrough, Paul Dirac famously noted that the underlying physical laws necessary for much of physics and all of chemistry are now completely known. However, he also pointed out that the exact application of these laws leads to equations that are much too complicated to solve^3^. This paradigm of theoretical chemistry and physics is prevalent until today. In particular, the exponential growth of the computational complexity of the many-electron problem with system size still makes an exact solution of the electronic Schrödinger equation for more than a few atoms impossible. As a consequence, a hierarchy of increasingly accurate methods that is capable of producing reference results at the expense of tractable yet high computational cost has emerged. These reference results are pivotal in order to develop, assess, and further improve computationally more efficient but in general less accurate approximations. In this context, a prime example was the numerical prediction of highly accurate ground state energies for the uniform electron gas using diffusion Monte Carlo methods, leveraging the development of approximate exchange-correlation functionals that ultimately led to the breakthrough of density functional theory in computational materials science during the last decades^4–6^.

At present, quantum mechanical many-electron calculations of systems containing more than 100 atoms have become possible thanks to methodological developments and considerable growth in computing power. These methodological improvements are often based on taking advantage of the relative nearsightedness of many-electron correlation effects^7–9^. In this manner, the scaling of the computational complexity with respect to system size can be lowered. However, recently several works^10–13^ showed that there exist alarming discrepancies between predicted interaction energies for large molecules when using two of the most widely-trusted highly accurate many-electron theories: DMC and CCSD(T), which stand for diffusion Monte Carlo and coupled-cluster theory using single, double, and perturbative triple particle-hole excitation operators, respectively. These observations are a source of great concern in the electronic structure theory community because, in the case of noncovalent interactions between molecular complexes, both CCSD(T) and DMC are considered highly reliable benchmark methods^14–16^. Furthermore, the observed discrepancies are large enough to cause qualitative differences in calculated properties of materials, which can have scientific and technological implications. For example, accurate crystal structure predictions are crucial in drug design^17–19^. Similarly, reliable reference methods are essential for discovering and designing new functional materials for applications such as renewable energy storage and conversion, including catalysis, or solar cells^20–22^. Finally, as machine learning increasingly pervades all areas of computational first-principles physics, the accuracy of these reference methods, which provide the training data, becomes even more critical^23–25^.

In the following, we analyze a set of large molecular systems where large discrepancies between approximated versions of DMC and CCSD(T) were observed^10,11^. Importantly, a direct experimental measurement of the computed interaction energies of these systems is complicated and prone to significant uncertainties. Therefore, it is an open challenge to identify the origin of the observed deviations for the employed highly accurate yet approximate theoretical approaches.

## Results

We present an approach which allows us to unambiguously test if the employed approximations for DMC and CCSD(T) cause the puzzling discrepancies between their predictions. In particular, our methodology exhibits three striking advantages. Firstly, due to its efficient and massive computational parallelization, we omit any local correlation approximation, as was employed for the CCSD(T) calculations in refs. ^11–13^. Secondly, we use a plane wave basis set to enable an unbiased assessment of the quality of previously employed tabulated atom-centered basis functions. Thirdly, we are able to study the influence of higher-order contributions to the many-electron perturbation expansion beyond CCSD(T) theory for large molecular complexes.

In order to demonstrate the reliability of our plane wave basis approach, we first investigate the parallel displaced benzene dimer as a benchmark. We find that our approach effectively addresses the challenges of noncovalent interactions between large molecules, combining the compactness and systematic improvability of natural orbitals without near-linear dependencies that plague atom-centered Gaussian basis sets for densely packed structures. As discussed in the supplementary information, our computed CCSD(T) interaction energies for the parallel displaced benzene dimer are in excellent agreement with Gaussian basis set results. Our fully converged estimate of the CCSD(T) interaction energy is -2.62kcal/mol, which is in excellent agreement with results obtained using Gaussian basis sets of -2.70 kcal/mol. We note that these results have been extrapolated to the complete basis set limit and in the case of our plane wave basis calculations also to the infinite box size limit.

Next, we turn to the parallel displaced coronene dimer (C2C2PD) interaction energy, where significant discrepancies between DMC and CCSD(T) have been observed^10,11^. A comparison to published second-order Møller-Plesset perturbation theory (MP2) interaction energies in Table 1 for C2C2PD reveals that our plane wave approach yields reliable results. Computational details about the basis set convergence are summarized in the supplementary information. As shown in Table 1, our canonical CCSD(T) estimates for the parallel displaced coronene dimer align closely with domain-based local pair-natural orbital (DLPNO-CCSD(T)), explicitly correlated pair-natural orbital (PNO-LCCSD(T)-F12) and local natural orbital (LNO-CCSD(T)) results, ruling out basis set incompleteness and local approximation errors as sources of discrepancies with DMC findings. Although our CCSD(T) calculations require about 100k CPU hours, the main purpose of these calculations is to serve as a valuable reference for computationally faster techniques whose approximations need to be checked carefully. It is noteworthy that the CCSD(T) interaction energy contains a large (T) contribution of about  −8 kcal/mol, indicating that the correct treatment of triple particle-hole excitation effects for the electronic correlation plays a crucial role.

**Table 1:** Calculated interaction energies in kcal/mol of the parallel displaced coronene dimer (C2C2PD)

Theory	Interaction energy	Ref.
MP2	− 38.5 ± 0.5	this work
MP2	− 38.1	Ref. ^13^
CCSD	− 13.4 ± 0.5	this work
CCSD(T)	− 21.1 ± 0.5	this work
LNO-CCSD(T)	− 20.6 ± 0.6	Ref. ^11^
DLPNO-CCSD(T_0_)	− 20.9 ± 0.4	Ref. ^13^
PNO-LCCSD(T)-F12	− 20.0	Ref. ^45^
DMC	− 18.1(8)	Ref. ^11^
DMC	− 17.5(14)	Ref. ^10^
CCSD(cT)	− 19.3 ± 0.5	this work

Results have been obtained at different levels of theory including MP2, CCSD, CCSD(T), CCSD(cT) and DMC. The uncertainty of the referenced DMC, LNO and DLPNO results are taken from the corresponding reference. The uncertainty of this work’s results are dominated by the remaining basis set error and the uncertainty of the box size extrapolation. Abbreviations: MP2, Second-order Møller-Plesset perturbation theory; CCSD, coupled cluster singles and doubles theory; (T) and (cT) refer to different perturbative triples methods described in the article; DMC, diffusion quantum Monte Carlo; LNO and DLPNO refer to different local approximations referenced in the article.

### All that glitters is not gold: overcorrelation in CCSD(T)

Having ruled out errors from local approximations and incomplete basis sets for the parallel displaced coronene dimer (C2C2PD), we seek to assess the (T) approximation, which contributes significantly to the interaction energy of C2C2PD. In passing we anticipate that the (T) contribution to the interaction energy is also relatively large for all other systems with a significant discrepancy reported in ref. ^11^ (see supplementary information).

The (T) approximation was introduced in the seminal work by Raghavachari et al.^26^. Since then, it has become one of the most widely-used benchmark methods—sometimes referred to as the ‘gold standard’ of molecular quantum chemistry–for weakly correlated systems. However, we argue that the partly significantly too strong interaction energies in CCSD(T) theory are caused by the employed truncation of the approximation of the triple particle-hole excitation operator. These shortcomings are comparable to the issue of too strong interaction energies from truncated perturbation theories for systems with large polarizabilities, as discussed by Nguyen et al.^27^. As can be observed for the coronene dimer in Table 1, second-order Møller-Plesset perturbation theory (MP2)—a truncated pertubation theory—exhibits this overestimation of the interaction energy. In the extreme case of an infinite polarizability, as it occurs in metallic systems, MP2 and CCSD(T) even yield divergent correlation energies in the thermodynamic limit, which is referred to as infrared catastrophe^28,29^. In contrast, a resummation of certain terms to infinite order can yield interaction energies with an accuracy that is less dependent on the polarizability. Prominent examples for such approaches include the CCSD theory as well as the random-phase approximation. We have recently presented a method, denoted as CCSD(cT), that averts the infrared catastrophe of CCSD(T) by including selected higher-order terms in the triples amplitude approximation without significantly increasing the computational complexity^29^.

#### Understanding the discrepancy

For the present work it is important to note that the main difference between CCSD(cT) and CCSD(T) theory originates from the employed approximation to the triple particle-hole excitation amplitudes. The triple amplitudes of the (cT) approximation are given in diagrammatic and algebraic form by^29^1where $\hat{V}$ and ${\hat{T}}_{2}$ stand for the Coulomb interaction and the double particle-hole excitation operator, respectively. For brevity, the contributions from the single excitation operator are not included and only one additional ‘direct’ diagram is depicted. In here, ${\Delta }_{abc}^{ijk}={\varepsilon }_{i}+{\varepsilon }_{j}+{\varepsilon }_{k}-{\varepsilon }_{a}-{\varepsilon }_{b}-{\varepsilon }_{c}$, with *ε*’s being one-electron HF energies. The bra- and ket-states correspond to a triple excited and reference state, respectively. The (T) approximation disregards the term $[[\hat{V},{\hat{T}}_{2}],{\hat{T}}_{2}]$, which is included in (cT) and also occurs in full CCSDT theory. This term effectively screens the bare Coulomb interaction of the $[\hat{V},{\hat{T}}_{2}]$ term and has an opposite sign, making it crucial for systems with large polarizability. However, for small and weakly polarizible systems the $[[\hat{V},{\hat{T}}_{2}],{\hat{T}}_{2}]$ contribution is small, making the (T) and (cT) approximation agree, as it was already shown for a set of small molecules^29^.

**Fig. 1:**
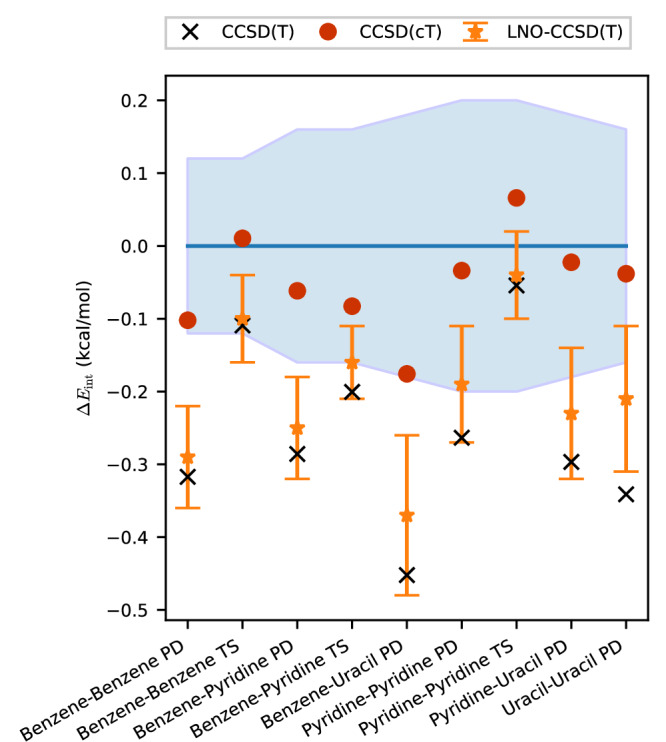
Deviations of coupled cluster results from diffusion quantum Monte Carlo (DMC) results for noncovalently bound dimers with up to 24 atoms. The dimers are in parallel displaced (PD) or T-shaped (TS) configurations. The CCSD(T) and CCSD(cT) values are calculated complete basis set (CBS) estimates obtained from basis set extrapolation using aug-cc-pVTZ and aug-cc-pVQZ basis sets^46,47^ (details in the supplementary information). The LNO-CCSD(T) and DMC results as well as their uncertainties are taken from ref. ^11^. The uncertainty of LNO-CCSD(T) is shown by the error bars. The uncertainty of DMC is shown by the blue area. Source data are provided as a Source Data file.

We now demonstrate that using CCSD(cT) instead of CCSD(T) theory restores excellent agreement for noncovalent interaction energies with DMC findings. First, we consider again the coronene dimer. Table 1 shows that the binding energy for the coronene dimer calculated on the level of CCSD(cT) theory is by almost 2 kcal/mol closer to the DMC estimate compared to CCSD(T) theory, achieving chemical accuracy (1 kcal/mol) in comparison to DMC after subtracting error bars. Next, we investigate the accuracy of CCSD(T) and CCSD(cT) compared to DMC for noncovalent interactions in smaller molecules. To this end, we study a set of dimers containing up to 24 atoms that were also investigated in ref. ^11^. This gives us another opportunity to assess the effect of local approximations at the level of CCSD(T) theory. Figure 1 depicts the deviations of all computed interaction energies from DMC reference values taken from ref. ^11^. It should be noted that DMC references and differences to LNO-CCSD(T) interaction energies are shown with error bars^11^. Using our massive computational parallelization approach, we are able to add canonical CCSD(T) interaction energies extrapolated to the CBS limit to the comparison to DMC. For these relatively small molecules, we can employ sufficiently large basis sets, reducing the remaining uncertainty to ~0.01 kcal/mol (see supplementary information). Importantly, our canonical CCSD(T) results are in good agreement with LNO-CCSD(T) findings to within its error bars. The only minor exception is observed for the parallel displaced uracil dimer, where canonical CCSD(T) predicts a slightly stronger interaction. A comparison to DMC reveals that CCSD(T) theory predicts on average about 0.3 kcal/mol stronger interaction energies. Based on LNO-CCSD(T) and DMC data alone such a statement cannot be made due to the relatively large and mostly overlapping error bars. However, our well converged canonical CCSD(T) findings allow drawing such conclusions. Only for the T-shaped pyridine and benzene dimers, DMC and CCSD(T) binding energies agree to within the DMC errors. Note that these systems have a smaller (T) contribution to the intereaction energy, compared to the parallel displaced systems. All other systems exhibit small but significant discrepancies between CCSD(T) and DMC results, which is consistent with the even larger discrepancies reported for the larger molecules in ref. ^11^. Similar to our findings for the coronene dimer reported in Table 1, Fig. 1 shows that CCSD(cT) interaction energies agree significantly better with DMC values than their CCSD(T) counterparts.

**Fig. 2:**
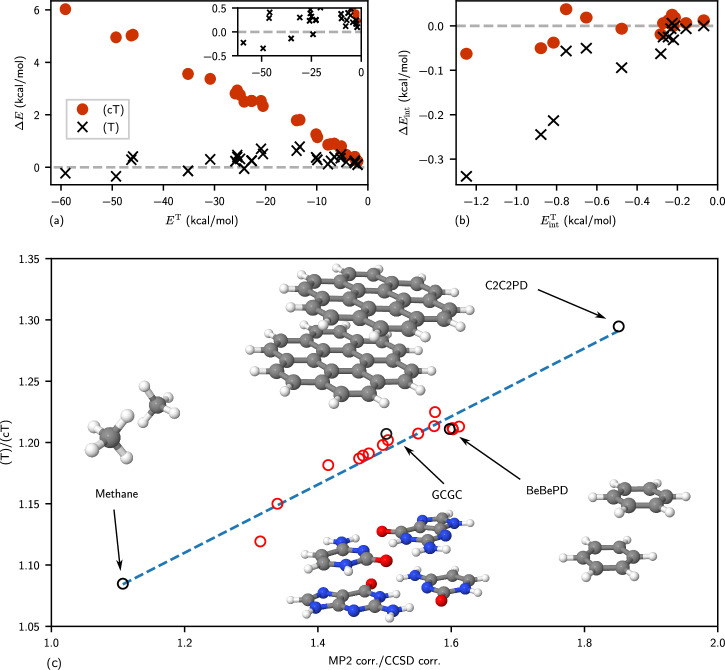
Comparison between the full triples and the perturbative triples approaches, (cT) and (T) for a set of molecules contained in the S22 data set^48^. The total triples correlation energy contribution *E*^*T*^ on the x-axis is compared to both differences between the (T), (cT) correlation energy contributions and *E*^*T*^ for (**a**) total energies (*ΔE*) and (**b**) interaction energies (*ΔE*_int_). The inset in (**a**) depicts a zoom of the figure. The abscissa is represented by a gray dashed line. Source data are provided as a Source Data file. **c** Correlation between the ratio of (T) and (cT) with the ratio of the MP2 and CCSD correlation energy contributions to interaction energies of a set of dispersion-dominated complexes from the S22, L7 and S66 benchmark datasets^48–50^. The blue dashed line is a linear fit of the data points. Selected cases are labeled and visualized: Methane dimer, GCGC (guaninecytosine tetramer), BeBePD (benzene dimer parallel displaced), C2C2PD (coronene dimer parallel displaced). All the data are available in the supplementary information. Source data are provided as a Source Data file.

Given the good agreement between DMC and CCSD(cT) for the systems studied above, an important question to ask is if CCSD(cT) is really more accurate than CCSD(T) for noncovalent interaction energies? To answer this question we compare interaction energies of both approaches to higher-level CC methods for complexes from the S22 data set. As can be observed in Fig. 2, we find that while (T) is in good agreement with T for total energies, it overestimates interaction energies. Here T stands for the triples contribution to the correlation energy, *E*^T^ = *E*^CCSDT^ − *E*^CCSD^. While (cT) total energies do not match the accuracy of (T) when compared to T, this is a secondary consideration in the present work. Our primary focus is on interaction energies, where we demonstrate that (cT) significantly improves the accuracy compared to (T). As shown in Fig. 2, (cT) closely matches the T interaction energies, indicating its superior accuracy for weakly bound complexes. This effect is particularly strong for interaction energies with large triples correlation contributions. Here, additional triples contributions, neglected in the (T) model, are required to resolve the systematic overestimation of (T). In the supplementary information we present a more detailed analysis, which indicates that the most important contributions indeed come from fifth-order ring diagram contributions depicted in Eq. (1).

### Estimating the overcorrelation of (T) for weak interactions

In summary, we have demonstrated that CCSD(cT) theory achieves excellent agreement for noncovalent interaction energies between molecular complexes compared to DMC and CCSDT theory. However, we stress that the CC series of methods (CCSD, CCSDT and CCSDTQ) is observed to yield monotonic and rapidly converging interaction energies for small and weakly bound complexes^30^. Based on this knowledge, we emphasize that the Q contribution to the interaction energies can be expected to be smaller than its T counterpart, but could possibly yield a significant contribution. Indeed, this is part of the reason for the success of the CCSD(T) approximation for very small molecules, where CCSD(T) is often fortuitously closer to CCSDTQ than CCSDT^30^. Here, we argue that this error cancellation no longer functions in the case of large molecular complexes involving strongly polarizable systems such as C2C2PD, C3GC and C60@[6]CPPA. A similar problem is known to occur in Møller-Plesset theory, where the truncation of the perturbation series also leads to significantly too strong interaction energies for systems with large polarizability, although MP2 yields relatively accurate interaction energies for systems with an intermediate polarizability^27^. To quantify and support the statements above, Fig. 2c illustrates that there exists a correlation between the ratio of (T) and (cT) with the ratio of the MP2 and CCSD correlation energy contributions to the interaction energies of all studied molecules in this work with dispersion-dominated interactions. This demonstrates that (T) exhibits a tendency to overestimate the absolute binding energy in a similar manner as MP2 for more polarizable systems. Although an overestimation of the (T) binding energy contribution compared to its (cT) counterpart by about 10% might yield a fortuitously better agreement between CCSD(T) and CCSDTQ, we argue that 20–30% overestimation is expected to yield significantly too strong interaction energies. For example, the values of (T)/(cT) for the Benzene-Benzene PD and coronene dimer are 1.2 and 1.3, respectively.

Having demonstrated and explained the reasons for the overestimation of absolute interaction energies on the level of CCSD(T) theory for small molecules with up to 24 atoms and the C2C2PD system, we now want to turn to the discussion of the remaining large molecular complexes where significant absolute discrepancies between DMC and CCSD(T) have been observed. These systems include C3GC from the L7 data set and the C60@[6]CPPA buckyball-ring. Here, substantial differences in the binding energies of 2.2 kcal/mol and 7.6 kcal/mol were reported, respectively after subtracting error bars. Although CCSD(cT) calculations for systems of that size are currently not feasible using our approach, we now introduce a simplified model that allows us to estimate the change in interaction energies from CCSD(T) to CCSD(cT) in an approximate manner. Given the linear trend between the different correlation energy contributions to the interaction energy depicted in Fig. 2c, it is possible to estimate the (cT) contribution for systems where only MP2, CCSD and (T) are known. These numbers can be calculated using a computationally efficient LNO-CCSD(T) implementation^31–36^. Results computed in this manner are denoted as CCSD(cT)-fit. Details about this procedure and the corresponding error estimates are provided in the supplementary information. Furthermore, the supplementary information includes a detailed justification of the CCSD(cT)-fit approach, which is based on extrapolation methods used to estimate the interaction energy between molecules and substrates modeled by planar molecules.

Table 2 gives our estimated CCSD(cT) interaction energies in comparison to CCSD(T) and DMC findings for seven large molecular complexes. A comparison between CCSD(cT)-fit and the explicitly calculated CCSD(cT) results for GGG, GCGC and C2C2PD shows that the linear regression model is sufficiently reliable for the systems studied in this work. Moreover, we have computed basis set converged (cT)-(T) estimates for C3A and PHE, which have been added to the LNO-CCSD(T) estimates to approximate CCSD(cT). These values and the agreement between CCSD(cT) and CCSD(cT)-fit explicitly confirm the CCSD(cT)-fit approach.

**Table 2:** Comparison of the interaction energy for large molecular complexes in kcal/mol as calculated by different levels of theory

System	CCSD(T)	LNO-CCSD(T)^11^	CCSD(cT)	CCSD(cT)-fit	DMC^11^	DMC^10^
GGG	− 1.5 ± 0.5	− 2.1 ± 0.2	− 1.2 ± 0.5	− 1.8 ± 0.2	− 1.5(6)	− 2.0(8)
GCGC	− 13.1 ± 0.5	− 13.6 ± 0.4	− 12.5 ± 0.5	− 12.8 ± 0.5	− 12.4(8)	− 10.6(12)
C2C2PD	− 21.1 ± 0.5	− 20.6 ± 0.6	− 19.3 ± 0.5	− 18.9 ± 0.7	− 18.1(8)	− 17.5(14)
C3A		− 16.5 ± 0.8	− 16.0 ± 0.8^*^	− 15.3 ± 0.9	− 15.0(10)	− 16.6(18)
PHE		− 25.4 ± 0.2	− 25.1 ± 0.2^*^	− 25.0 ± 0.2	− 26.5(13)	− 24.9(12)
C3GC		− 28.7 ± 1.0		− 26.7 ± 1.1	− 24.2(13)	− 25.1(18)
C_60_@[6]CPPA		− 41.7 ± 1.7		− 36.9 ± 2.3	− 31.1(14)	

Showcasing partially large discrepancies between CCSD(T) and DMC on the one hand, and an excellent agreement between CCSD(cT) and DMC results for complexes up to the 100-atom scale on the other hand. CCSD(T) and CCSD(cT) results are obtained using our plane wave approach. Values for CCSD(cT)-fit are taken from the LNO-CCSD(T) results of^11^ and corrected by an estimate for *Δ* = (T) − (cT) obtained from a fit. CCSD(cT) estimates with a * were estimated by computing *Δ* separately and adding it to the LNO-CCSD(T) values. Details of the procedures can be found in the supplementary information. Abbreviations: CCSD, coupled cluster singles and doubles theory; (T) and (cT) refer to different perturbative triples methods described in the article; LNO refers to a local approximation referenced in the article; DMC, diffusion quantum Monte Carlo; GGG, Guanine trimer; GCGC, Guanine-Cytosine tetramer; C2C2PD, Coronene dimer parallel displaced; C3A, Circumcoronene adenine; PHE, Phenylalanine residues trimer; C3GC, Circumcoronene Guanine-Cytosine.

For comparison Table 2 also summarizes the DMC interaction energies from refs. ^10^ and ^11^, which agree to within at least 1 kcal/mol for GGG, C2C2PD, PHE and C3GC. For the remaining systems the DMC estimates show a larger discrepancy and for C60@[6]CPPA only one DMC estimate is available. Although the DMC binding energies have overlapping error bars, the remaining uncertainties are relatively large, illustrating that obtaining highly accurate interaction energies for these large molecules is also challenging for DMC.

As already discussed in ref. ^11^, CCSD(T) interaction energies listed in Table 2 exhibit large discrepancies compared to DMC for C2C2PD, C3GC and C60@[6]CPPA. In contrast, CCSD(cT)-fit resolves these discrepancies for all systems on the hundred-atom scale, achieving excellent agreement with DMC estimates of Al-Hamdani and Nagy et al.^11^ to within chemical accuracy (1 kcal/mol) after subtracting the error bars. Even for C60@[6]CPPA, which contains 132 atoms, a discrepancy of only 2.1 kcal/mol remains, although the error bar of CCSD(cT)-fit is relatively large in this case. We argue that the remaining discrepancies are potentially caused by uncertainties in DMC, CCSD(cT)-fit and the underlying LNO-CCSD(T) calculations. It should be noted that the error bars of LNO-CCSD(T) interaction energies are in some cases underestimated, as exemplified for the Uracil-Uracil PD dimer by the comparison between canonical CCSD(T) and LNO-CCSD(T) interaction energies shown in Fig. 1. Furthermore, the DMC interaction energy of C60@[6]CPPA has not yet been verified independently using a different DMC implementation as it was done for all other systems listed in Table 2. We also stress that in some cases the differences between the DMC estimates are larger than their respective error bars.

## Discussion

Our work unequivocally demonstrates that, due to the employed truncation of the many-body perturbation series expansion, one of the most widely-used and accurate quantum chemistry approaches—CCSD(T) theory—in certain cases binds noncovalently interacting large molecular complexes too strongly. Our findings show that a simple yet efficient modification denoted as CCSD(cT) remedies these shortcomings, enabling highly reliable benchmark calculations of large molecular complexes on the hundred-atom scale that play a crucial role in scientific and technological problems, for example, drug design and surface science. We stress that the more accurate CCSD(cT) approximation can directly be transferred to computationally efficient low-scaling and local correlation approaches, which will substantially advance the applications of theoretical chemistry as well as physics in all areas of computational materials science where highly accurate benchmark results are urgently needed. We are witnessing an unremitting expansion of the frontiers of accurate electronic structure theories to ever larger systems which when combined with machine-learning techniques, has the potential to transform the paradigm of modern computational materials science.

## Methods

### Benchmark systems

We considered representative subsets of the L7, S22, and S66 benchmark sets of noncovalent interactions. These structures can be found in the begdb database^37^. Nine systems from the S66 set were selected to obtain extrapolated complete basis set (CBS) estimates, while 18 systems from the S22 set were studied at the CCSDT level of theory with modest basis sets. Furthermore, two additional groups of systems, parallel displaced polycyclic aromatic hydrocarbons, and single adenine on top of a polycyclic aromatic hydrocarbon have been studied. The benzene-benzene structures are taken from the S66 set, and the coronene dimer structure is taken from the L7 test database. All other structures not included in the abovementioned data sets, were obtained by geometry optimization using the NWChem^38^ version 7.0.0 with the TPSS functional and def2-tzvp basis sets. Furthermore, we include the employed structures in the SI as xyz-files.

### Gaussian basis set calculations

For the S66 systems, we employed Dunning’s augmented correlation-consistent basis sets (aug-cc-pVXZ, X = T, Q, 5). CBS estimates were obtained using standard two-point extrapolation, denoted as [34] (triple-*ζ* and quadruple-*ζ*) and [45] (quadruple-*ζ* and quintuple-*ζ*). Interaction energies were defined with and without counterpoise (CP) correction. Canonical MP2, CCSD, and CCSD(T) correlation energies were obtained with the MRCC package, interfaced to our Cc4s code. In all MRCC calculations, the program’s default resolution-of-identity (RI) auxiliary basis sets were employed for both Hartree-Fock and post-HF correlation steps. We use the MRCC version released on March 18, 2022^31–33^. Based on basis set convergence tests, the aug-cc-pVQZ basis set provides essentially converged Hartree-Fock energies, while the [34] extrapolation of MP2 correlation energies closely approximates the [45] CBS estimate. All of these observations hold for the counterpoise-corrected results. Given that CCSD(T) correlation energies typically converge faster with respect to basis set size than MP2, these findings justify using the [34] approach as a reliable estimate of the CBS limit for the S66 systems shown in Fig. 1.

For the S22 set, Hartree-Fock energies were computed with NWChem^38^ version 7.0.0 using the cc-pVDZ basis, while post-Hartree-Fock methods (MP2, CCSD, CCSDT) were carried out via Cc4s. In this case, counterpoise corrections were not applied, and the reported interaction energies are based directly on the raw supermolecular results.

For the parallel displaced polycyclic aromatic hyrdocarbons, and single adenine on top of a polycyclic aromatic hydrocarbon systems we employed aug-cc-pVTZ basis sets using MRCC. Results are given using the counterpoise correction. For the coupled-cluster calculations we used frozen natural orbitals (FNOs)^39^ with *X* = 8 − 12 virtual orbitals per occupied orbital. CCSD energies are corrected using the Delta-MP2 approach, *E*_CCSD_ = *E*_CCSD_(*X*) + *E*_MP2_(aug − cc − pVTZ) − *E*_MP2_(*X*), and the (T) and (cT) energies are corrected using the (T*) method, *E*_(T*)_ = *E*_(T)_(*X*) ⋅ *E*_MP2_(aug − cc − pVTZ)/*E*_MP2_(*X*)^40,41^.

Finally, we have calculated CBS estimates for C3A and PHE using Gaussian type orbitals with MRCC. Therefore, we took counterpoise corrected results using aug-cc-pVQZ basis set. It was shown that this choice is already sufficient for very accurate CBS estimates^11^. In these calculations we employ approximative FNOs: 6 and 10 for C3A and 8 and 10 for PHE, respectively. For the final CCSD energies, we once more use Delta-MP2 corrected values, defined above. For these numbers we use MP2 CBS estimates using canconical MP2 calculations, employing [45] extrapolation. Similarly (T*) correction is used, again using [45] extrapolated values for the MP2 CBS estimate.

### Plane-wave workflow

For larger molecular complexes, we employed a plane-wave workflow implemented in a development version of VASP 6.3 and Cc4s. Simulation cells were enlarged systematically to remove spurious interactions between periodic images, and plane-wave cutoffs were converged at 700 eV based on MP2 tests. Canonical Hartree-Fock orbitals were computed and subsequently transformed to approximate MP2 natural orbitals^39^. Truncated sets of natural orbitals, characterized by the ratio of virtual to occupied states (*X* = *N*_*v*_/*N*_*o*_), were used both for efficient MP2 CBS corrections and as input for coupled-cluster calculations. Coulomb integrals were factorized using a singular-value decomposition of the auxiliary basis^42^, allowing for substantial reduction of the auxiliary dimension without loss of accuracy.

Final CCSD, CCSD(T), and CCSD(cT) calculations were performed with Cc4s on up to 50 compute nodes (128 cores each), exploiting the code’s massively parallel implementation.

## Supplementary information

Supplementary Information

Description of Additional Supplementary Files

Supplementary Data 1

Transparent Peer Review file

## Source data

Source Data

## Data Availability

Additional data supporting the findings of this study are available within the supplementary information. The supplementary data file contains molecular structures in xyz-format used for calculations presented in S6. Source data are provided with this paper.

## Code availability

The Cc4s code^43^ developed and used for the presented post-HF calculations can be downloaded from(10.5281/zenodo.16762158^44^). A documentation and more information about the code can be found in ref. ^43^. For the underlying HF calculations we employed different codes that are referenced in the Methods section.
