# Supplementary material for: Understanding discrepancies in noncovalent interaction energies from wavefunction theories for large molecules
Source: Nat Commun. 2025 Oct 14;16:9108. doi: 10.1038/s41467-025-64104-8 (PMC12521354; doi:10.1038/s41467-025-64104-8)
Supplement: Supplementary file 1 — Supplementary Information [file 41467_2025_64104_MOESM1_ESM.pdf]

Supplementary information for: Understanding Discrepancies in noncovalent interaction energies from Wavefunction Theories for Large Molecules

Tobias Schäfer,* Andreas Irmler,† Alejandro Gallo, and Andreas Grüneis‡
Institute for Theoretical Physics, TU Wien,
Wiedner Hauptstraße 8-10/136, 1040 Vienna, Austria

CONTENTS

S1. Supplementary information notes 1

S2. Selected S66 systems - canonical CBS estimates 2

S3. Selected S22 systems - CCSDT using cc-pVDZ basis sets 4

S4. The plane wave based workflow 6
Benzene dimer (parallel displaced) 6
Coronene dimer (parallel displaced) 6

S5. Estimating CCSD(cT)-fit and its uncertainty 8

S6. Justification of CCSD(cT)-fit 9

S7. Estimating CCSD(cT) for PHE and C3A 10

S8. S22 data set results for perturbative triples methods 11

S9. Overview of Employed and Newly Generated Structures 11
Pyrene dimer 13
Adenine on Benzene 14
Adenine on Naphthalene 15
Adenine on Pyrene 16
Adenine on Coronene 17

Supplementary References 18

S1. SUPPLEMENTARY INFORMATION NOTES

- The CBS estimates for benzene-benzene PD (used in Fig. 1) and the Gaussian basis CCSD(T) estimate of -2.70 kcal/mol are presented in section S2. Furthermore, the CBS estimates for CCSD(T) and CCSD(cT) shown in Fig. 1 can be found in section S2. These numbers are from counterpoise corrected calculations from AVQZ Hartree–Fock calculations together with correlation calculations using a [34] extrapolation. All values are provided in Table S I
- Results depicted in Fig. 3 for molecules contained in the S22 data set on the level of T, (T) and (cT) theory are summarized in S3
- The plane wave approach used for the results in Table 1, Fig. 3, and Table 2 is explained section S4
- Results for (T) energy contributions of the large molecules from the L7 set and C60[6]CPPA can be found in Table S V
- The CCSD(cT)-fit is explained in section S5 and a justification is given in section S6
- Data used in Fig. 3 is summarized in Table S IV

* tobias.schaefer@tuwien.ac.at
† andreas.irmler@tuwien.ac.at
‡ andreas.grueneis@tuwien.ac.at

S2. SELECTED S66 SYSTEMS - CANONICAL CBS ESTIMATES

Here we provide highly accurate CBS estimates using Gaussian type orbitals for 9 molecules from the S66 test set. For Gaussian type orbitals there exists a well established strategy to reach the complete basis set limit (CBS). We use Dunning's correlation consistent basis sets of type aug-cc-pVXZ (AVXZ), where X refers to the cardinal number of the basis set. In this work, we employ X = T, Q, and 5. CBS estimates in post-Hartree–Fock methods are obtained by a two point extrapolation assuming a X^{-3} convergence. The extrapolation using AVTZ and AVQZ is denoted as [34], whereas [45] employs the basis sets AVQZ and AV5Z.

Table S I shows the convergence of the interaction energies of the studied molecules with respect to the employed basis set. Results are given for Hartree–Fock, as well as for MP2, CCSD, (T) and the (cT) contribution. Correlation energies were obtained using the `MRCC` [1–4] interfaced to our `Cc4s` code [5].

For these systems, we are able to calculate canonical MP2 results with the AVTZ, AVQZ, and AV5Z basis. One can see that the counterpoise corrected (CP) and the uncorrected (NC) results agree well when the largest available basis set is used. For HF the CP and NC with the AV5Z deviate only by 0.009 kcal/mol or 0.014 kcal/mol for root mean square deviation (rms) and maximal deviation (max), respectively. For MP2 the best available estimate for the complete basis set would be the [45] extrapolation. Here CP and NC deviate by 0.076 and 0.117 kcal/mol rms and max, respectively. It can be seen that results from smaller basis sets are significantly better for CP then for the uncorrected case. For HF the CP corrected results using AVQZ and AV5Z are for all intents and purposes identical, with a maximum deviation of 0.002 kcal/mol. In the NC case AVQZ and AV5Z differ by 0.064 and 0.109 kcal/mol for rms and max, respectively. The same can be observed for MP2, here for the CP results the [34] result is already very close to the [45] value, namely 0.007 and 0.017 kcal/mol for rms and max, respectively. NC results show a larger deviation of 0.112 and 0.250 kcal/mol for rms and max, respectively.

These results allow to conclude that both, AVQZ for HF and [34] extrapolation for MP2, are sufficiently accurate for the given set of systems.

Now we can turn to the CCSD(T) correlation energies. As the BSIE of CCSD(T) is known to be equal and mostly even smaller than in MP2, the provided results obtained from [34] extrapolation are expected to be very close to the CBS. Consequently, the expected deviations from the CBS limit are in the order of 0.01 kcal/mol or lower. These findings are in accordance with canonical CBS estimates from Nagy et al. [6] for the same system using slightly smaller basis sets. Further CBS estimates for these systems are obtained from Ma and Werner [7] and Kesharwani et al. [8].

Table S I: Interaction energies in kcal/mol of the studied molecular systems with different Gaussian basis sets. Shown is the Hartree–Fock energy contribution as well as the canonical correlation energies for MP2, CCSD, (T), and (cT). Both counterpoise (CP) corrected results as well as results without CP are presented.

		CP corrected					CP uncorrected				
	Method	AVTZ	AVQZ	AV5Z	[34]	[45]	AVTZ	AVQZ	AV5Z	[34]	[45]
Pyridine-pyridine PD	HF	3.336	3.332	3.331	-	-	3.112	3.273	3.324	-	-
	MP2 corr.	-9.100	-9.238	-9.287	-9.339	-9.339	-10.203	-9.717	-9.503	-9.362	-9.279
	CCSD corr.	-5.673	-5.743	-	-5.794	-	-6.597	-6.061	-	-5.670	-
	(T)	-1.254	-1.286	-	-1.310	-	-1.341	-1.321	-	-1.307	-
	(cT)	-1.029	-1.059	-	-1.080	-	-1.114	-1.093	-	-1.078	-
Pyridine-pyridine TS	HF	0.869	0.867	0.867	-	-	0.703	0.822	0.861	-	-
	MP2 corr.	-5.054	-5.172	-5.213	-5.258	-5.255	-6.016	-5.581	-5.400	-5.263	-5.211
	CCSD corr.	-3.457	-3.538	-	-3.597	-	-4.267	-4.267	-	-3.479	-
	(T)	-0.727	-0.747	-	-0.762	-	-0.801	-0.776	-	-0.758	-
	(cT)	-0.609	-0.628	-	-0.642	-	-0.682	-0.657	-	-0.639	-
Benzene-pyridine PD	HF	3.621	3.619	3.618	-	-	3.395	3.559	3.610	-	-
	MP2 corr.	-8.838	-8.962	-9.006	-9.052	-9.053	-9.991	-9.439	-9.223	-9.036	-8.996
	CCSD corr.	-5.552	-5.609	-	-5.651	-	-6.523	-5.925	-	-5.488	-
	(T)	-1.229	-1.260	-	-1.282	-	-1.320	-1.295	-	-1.277	-
	(cT)	-1.009	-1.038	-	-1.058	-	-1.098	-1.072	-	-1.054	-
Benzene-pyridine TS	HF	0.943	0.943	0.943	-	-	0.745	0.896	0.936	-	-
	MP2 corr.	-4.936	-5.042	-5.079	-5.119	-5.119	-6.016	-5.473	-5.273	-5.077	-5.063
	CCSD corr.	-3.369	-3.438	-	-3.488	-	-4.288	-3.725	-	-3.315	-
	(T)	-0.702	-0.721	-	-0.735	-	-0.786	-0.752	-	-0.728	-
	(cT)	-0.587	-0.605	-	-0.618	-	-0.668	-0.635	-	-0.611	-
Pyridine-uracil PD	HF	2.074	2.072	2.071	-	-	1.730	1.984	2.060	-	-
	MP2 corr.	-10.358	-10.556	-10.631	-10.701	-10.710	-11.935	-11.262	-10.945	-10.770	-10.613
	CCSD corr.	-6.097	-7.024	-	-7.110	-	-8.266	-7.516	-	-6.969	-
	(T)	-1.567	-1.607	-	-1.637	-	-1.692	-1.658	-	-1.634	-
	(cT)	-1.298	-1.335	-	-1.362	-	-1.421	-1.386	-	-1.360	-
Benzene-benzene PD	HF	3.964	3.961	3.960	-	-	3.739	3.901	3.952	-	-
	MP2 corr.	-8.479	-8.587	-8.625	-8.665	-8.666	-9.654	-9.052	-8.837	-8.613	-8.610
	CCSD corr.	-5.350	-5.392	-	-5.423	-	-6.345	-5.699	-	-5.227	-
	(T)	-1.183	-1.212	-	-1.234	-	-1.276	-1.247	-	-1.226	-
	(cT)	-0.973	-0.999	-	-1.019	-	-1.063	-1.033	-	-1.012	-
Benzene-benzene TS	HF	1.448	1.447	1.448	-	-	1.251	1.400	1.440	-	-
	MP2 corr.	-5.030	-5.125	-5.158	-5.194	-5.193	-6.158	-5.554	-5.352	-5.114	-5.141
	CCSD corr.	-3.419	-3.476	-	-3.518	-	-4.385	-3.762	-	-3.308	-
	(T)	-0.715	-0.734	-	-0.748	-	-0.802	-0.765	-	-0.738	-
	(cT)	-0.598	-0.616	-	-0.628	-	-0.682	-0.646	-	-0.620	-
Uracil-uracil PD	HF	0.388	0.379	0.377	-	-	-0.091	0.254	0.363	-	-
	MP2 corr.	-11.047	-11.323	-11.430	-11.525	-11.542	-13.152	-12.298	-11.872	-11.675	-11.425
	CCSD corr.	-7.802	-7.998	-	-8.142	-	-9.660	-8.700	-	-8.000	-
	(T)	-1.887	-1.936	-	-1.972	-	-2.052	-2.006	-	-1.972	-
	(cT)	-1.590	-1.636	-	-1.669	-	-1.751	-1.704	-	-1.670	-
Benzene-uracil PD	HF	3.444	3.443	3.442	-	-	3.070	3.352	3.431	-	-
	MP2 corr.	-10.658	-10.845	-10.914	-10.982	-10.987	-12.348	-11.566	-11.232	-10.996	-10.882
	CCSD corr.	-7.158	-7.260	-	-7.334	-	-8.620	-7.759	-	-7.131	-
	(T)	-1.598	-1.640	-	-1.670	-	-1.732	-1.692	-	-1.664	-
	(cT)	-1.327	-1.366	-	-1.394	-	-1.457	-1.417	-	-1.389	-

S3. SELECTED S22 SYSTEMS - CCSDT USING CC-PVDZ BASIS SETS

Here we provide the numerical data for 18 out of 22 molecular systems from the S22 benchmark set. Results are shown for canonical calculations using the cc-pVDZ basis set. The HF calculations were performed using NWCHEM [9] and for the post-HF methods we employed an interface to our Cc4s code [10]. Interaction energies shown in the manuscript are evaluated from data summarized in Table SII and are not counterpoise corrected.

Table S II: Results for molecular systems of the S22 dataset using the cc-pVDZ basis set in Hartree units. T correlation energy contribution is evaluated via CCSDT-CCSD. The *index* column denotes the identifier of the molecule in the S22 dataset. The *Type* column describes the type of system that has been computed, F1, F2 being the separate fragments, whereas Full denotes the whole system.

Index	System	Type	HF	MP2 corr.	CCSD corr.	(T)	(cT)	T
1	Ammonia dimer	F1	-56.195616	-0.186397	-0.202634	-0.003802	-0.003531	-0.004072
		F2	-56.195616	-0.186397	-0.202634	-0.003802	-0.003531	-0.004072
		Full	-112.396243	-0.375576	-0.407333	-0.007953	-0.007378	-0.008498
2	Water dimer	F1	-76.026603	-0.201874	-0.211441	-0.003051	-0.002844	-0.003214
		F2	-76.026710	-0.201741	-0.211310	-0.003041	-0.002835	-0.003204
		Full	-152.062536	-0.406176	-0.424477	-0.006434	-0.005987	-0.006755
3	Formicacid dimer	F1	-188.778390	-0.500591	-0.512818	-0.015288	-0.013906	-0.015736
		F2	-188.778390	-0.500591	-0.512818	-0.015288	-0.013906	-0.015736
		Full	-377.586256	-1.005708	-1.028426	-0.031867	-0.028964	-0.032677
4	Formamide dimer	F1	-168.945898	-0.483914	-0.502205	-0.015525	-0.014134	-0.016125
		F2	-168.945898	-0.483914	-0.502205	-0.015525	-0.014134	-0.016125
		Full	-337.916259	-0.972313	-1.007590	-0.032177	-0.029269	-0.033309
5	Uracil dimer H-bonded	F1	-412.502984	-1.187925	-1.216626	-0.046610	-0.042047	N/A
		F2	-412.502984	-1.187925	-1.216626	-0.046610	-0.042047	N/A
		Full	-825.036038	-2.380421	-2.436457	-0.094578	-0.085272	N/A
6	2-pyridoxine-2-aminopyridine complex	F1	-301.761197	-0.974961	-1.012909	-0.042603	-0.038232	-0.042742
		F2	-321.590611	-0.989790	-1.023629	-0.042430	-0.038222	-0.042937
		Full	-623.373341	-1.974487	-2.043169	-0.086892	-0.078069	-0.087354
7	Adeninethymine-Watson-Crick complex	F1	-451.544594	-1.333968	-1.374049	-0.052031	-0.046939	N/A
		F2	-464.558739	-1.443070	-1.470596	-0.064769	-0.057868	N/A
		Full	-916.124719	-2.786223	-2.851674	-0.118679	-0.106460	N/A
8	Methane dimer	F1	-40.198702	-0.161179	-0.184661	-0.003702	-0.003457	-0.004092
		F2	-40.198702	-0.161179	-0.184661	-0.003702	-0.003457	-0.004092
		Full	-80.396906	-0.323344	-0.370213	-0.007506	-0.007007	-0.008291
9	Ethene dimer	F1	-78.039915	-0.274850	-0.305009	-0.009796	-0.008999	-0.010426
		F2	-78.039915	-0.274850	-0.305009	-0.009796	-0.008999	-0.010426
		Full	-156.079311	-0.552564	-0.612197	-0.019981	-0.018347	-0.021230
10	Benzene-Methane complex	F1	-230.722189	-0.782664	-0.822131	-0.035812	-0.032184	-0.036208
		F2	-40.198646	-0.161040	-0.184473	-0.003686	-0.003442	-0.004074
		Full	-270.919640	-0.947566	-1.009489	-0.039973	-0.036041	-0.040713
11	Benzene dimer PD	F1	-230.722178	-0.782673	-0.822141	-0.035811	-0.032184	-0.036209
		F2	-230.722178	-0.782673	-0.822141	-0.035811	-0.032184	-0.036209
		Full	-461.437753	-1.578592	-1.652826	-0.073256	-0.065721	-0.073717
12	Pyrazine dimer	F1	-262.702440	-0.835880	-0.862195	-0.038626	-0.034562	-0.038548
		F2	-262.702461	-0.835842	-0.862162	-0.038618	-0.034555	-0.038541
		Full	-525.400639	-1.686240	-1.733417	-0.079039	-0.070599	-0.078490
13	Uracil dimer stack	F1	-412.504294	-1.187353	-1.216151	-0.046560	-0.042005	-0.047262
		F2	-412.504294	-1.187353	-1.216151	-0.046560	-0.042005	-0.047262
		Full	-825.012764	-2.389278	-2.442802	-0.095450	-0.085991	-0.096529
14	Indolebenzene complex stack	F1	-361.497850	-1.205004	-1.245524	-0.056259	-0.050375	-0.056044
		F2	-230.722148	-0.782743	-0.822212	-0.035828	-0.032199	-0.036226
		Full	-592.211534	-2.007890	-2.080529	-0.094617	-0.084658	-0.094257
15	Adeninethymine complex stack	F1	-464.559002	-1.443624	-1.471037	-0.064980	-0.058050	N/A
		F2	-451.546252	-1.332996	-1.373209	-0.051919	-0.046843	N/A
		Full	-916.106136	-2.801350	-2.861284	-0.120400	-0.107829	N/A
16	Etheneethyne complex	F1	-78.039902	-0.274863	-0.305019	-0.009799	-0.009002	-0.010429
		F2	-76.825504	-0.256064	-0.273040	-0.011148	-0.010133	-0.011540
		Full	-154.866735	-0.532540	-0.579048	-0.021214	-0.019372	-0.022218
17	Benzenewater complex	F1	-230.722144	-0.782775	-0.822245	-0.035841	-0.032210	-0.036238
		F2	-76.026578	-0.202033	-0.211581	-0.003066	-0.002859	-0.003230
		Full	-306.751679	-0.987721	-1.035750	-0.039306	-0.035417	-0.039820

Table S II: Results for molecular systems of the S22 dataset using the cc-pVDZ basis set in Hartree units. T correlation energy contribution is evaluated via CCSDT-CCSD. The *index* column denotes the identifier of the molecule in the S22 dataset. The *Type* column describes the type of system that has been computed, F1, F2 being the separate fragments, whereas Full denotes the whole system.

Index	System	Type	HF	MP2 corr.	CCSD corr.	(T)	(cT)	T
18	Benzeneammonia complex	F1	-230.722166	-0.782716	-0.822185	-0.035825	-0.032196	-0.036222
		F2	-56.195676	-0.186468	-0.202699	-0.003821	-0.003548	-0.004091
		Full	-286.918481	-0.972582	-1.027292	-0.040078	-0.036121	-0.040698
19	BenzeneHCN complex	F1	-230.722122	-0.782834	-0.822305	-0.035856	-0.032224	-0.036253
		F2	-92.881359	-0.287354	-0.295666	-0.012245	-0.011064	-0.012427
		Full	-323.607430	-1.074022	-1.120179	-0.048653	-0.043758	-0.049131
20	Benzene dimer TS	F1	-230.722171	-0.782715	-0.822184	-0.035823	-0.032194	-0.036220
		F2	-230.722165	-0.782736	-0.822205	-0.035829	-0.032200	-0.036227
		Full	-461.443197	-1.572462	-1.649192	-0.072557	-0.065161	-0.073207
21	Indolebenzene complex TS	F1	-230.722099	-0.782884	-0.822356	-0.035868	-0.032234	-0.036266
		F2	-361.497836	-1.205171	-1.245663	-0.056280	-0.050392	-0.056063
		Full	-592.221360	-1.997970	-2.074469	-0.093465	-0.083736	N/A
22	Phenol dimer	F1	-305.587135	-0.965780	-1.004421	-0.040920	-0.036806	-0.041289
		F2	-305.587007	-0.965693	-1.004388	-0.040908	-0.036797	-0.041281
		Full	-611.181100	-1.940136	-2.015029	-0.083048	-0.074649	-0.083654

S4. THE PLANE WAVE BASED WORKFLOW

In this section, the workflow to calculate interaction energies of large molecules in a plane wave basis under periodic boundary conditions is described. All calculations are performed with the Vienna Ab-Initio Simulation Package (VASP) [11] and the `Cc4s` [12] code.

1. A fixed box size and a plane-wave basis set size are chosen. The scheme is repeated for increasing box sizes to reach the infinite box size limit corresponding to the isolated molecule in the gas phase. The plane-wave basis set size was set via an energy cutoff of 700 eV (`ENCUT` flag in VASP). This choice resulted from a careful convergence test of the direct-MP2 correlation energy of the coronene dimer, achieving an accuracy well below 0.1 kcal/mol for a fixed box size.

2. The Hartree-Fock ground state is calculated using the given setting. Both the occupied as well as all unoccupied orbitals and orbital energies are stored.

3. Approximate natural orbitals at the MP2 level are calculated, as outlined in Ref. [13]. Natural orbitals are the eigenvectors of the one-electron reduced density matrix. The corresponding eigenvalues are called occupation numbers. Ordered by their occupation number, we truncated and recanonicalized the natural orbital basis by choosing a ratio N_v/N_o, where N_o is the number of occupied orbitals in the system and N_v is the number of chosen natural orbitals. The natural orbitals provide a basis which allows for a much more rapid convergence of the correlation energy with respect to N_v.

4. The MP2 energy is calculated in the CBS limit using the natural orbitals with $N_v/N_o = 200$. This is necessary for basis set correction schemes to estimate the CBS limit of the CCSD and (T) energies. The basis set correction scheme, called focal point correction, is described in Ref. [10].

5. To prepare the coupled cluster calculations a basis of $N_v/N_o = 15$ is chosen. All Coulomb integrals, V^{pq}_{sr}, needed by coupled cluster theory are computed using the expression

$$V^{pq}_{sr} = \sum_{F=1}^{N_F} {\Gamma^*}^{pF}_{s} \Gamma^{q}_{rF}, \tag{1}$$

   where p, q, r, s refer to occupied or virtual orbital indices. F denotes an auxiliary basis functions, obtained by a singular value decomposition outlined in Ref. [14]. Due to the large vacuum in the simulation cells, significant reductions of the auxiliary basis set size are possible without compromising the precision of computed correlation energies. The correlation energies are converged to within meV with respect to the size of the optimized auxiliary basis set.

6. The final coupled cluster calculations at the level of CCSD, CCSD(T), and CCSD(cT) are performed with our high-performance code called `Cc4s`. We employed up to 50 compute nodes with 128 cores each to run our massive computational parallelization approach.

Benzene dimer (parallel displaced)

We demonstrate that our plane wave basis approach works reliable for the study of noncovalent interactions between molecules and combines the best of two worlds: compactness and systematic improvability without linear dependencies. To this end we discuss the computed interaction energy of the parallel displaced benzene dimer on the level of CCSD(T) theory and compare to results from basis set converged Gaussian calculations. The total CCSD(T) energy is composed of three terms, the HF total energy, the CCSD correlation energy and the perturbative triples contribution which we denote as (T). Fig. S1(a) depicts the convergence of the CCSD and (T) correlation energy contributions to the computed CCSD(T) interaction energy for a fixed box size with respect to the number of basis functions (natural orbitals) per occupied state ($N_\mathrm{v}/N_\mathrm{o}$). We include a recently introduced correction to accelerate the convergence of correlation energies to the complete basis set limit (CBS) [10]. Our findings show that a basis set size of $N_\mathrm{v}/N_\mathrm{o} = 15$ suffices to achieve convergence to within a fraction of 0.1 kcal/mol. We employ this basis set to compute the CCSD(T) interaction energies and its Hartree–Fock (HF), CCSD and (T) correlation energy contributions for different simulation cell sizes. Fig. S1(b) shows that these contributions converge rapidly. Our fully converged estimate of the CCSD(T) interaction energy for the parallel displaced benzene dimer is −2.62 kcal/mol, which is in excellent agreement with results obtained using Gaussian basis sets of −2.70 kcal/mol.

Coronene dimer (parallel displaced)

Using the example of the coronene dimer (C2C2PD), Fig. S2 shows the dependence of the interaction energy on the box size. The interaction energy exhibits an exponential convergence of the form $a + b \cdot \mathrm{e}^{-cV^{1/3}}$, where V is the volume of the box. This behavior holds for both the HF and correlation contributions. The reliability of this extrapolation law is supported by RPA (random phase approximation) calculations of the correlation energy for volumes up to $\sim 5000\text{Å}^3$. This allows us to converge the interaction energy with a remaining uncertainty of less than 0.5 kcal/mol.

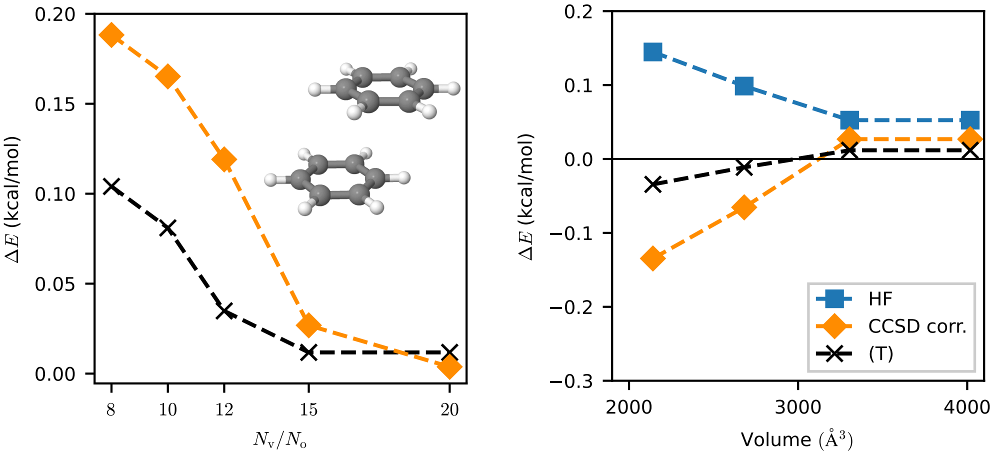

(a) (b)

Fig. S 1. **Convergence behavior of the interaction energy for the benzene parallel displaced dimer system.** ΔE is the difference between our plane wave based approach and the reference results from Gaussian basis calculations extrapolated to the CBS limit. **a,** shows the convergence with respect to the number of natural orbitals for a fixed volume of about 4018 Å^3. **b,** displays the convergence with respect to the volume of the simulation cell with a fixed number of natural orbitals of $N_v/N_o = 15$.

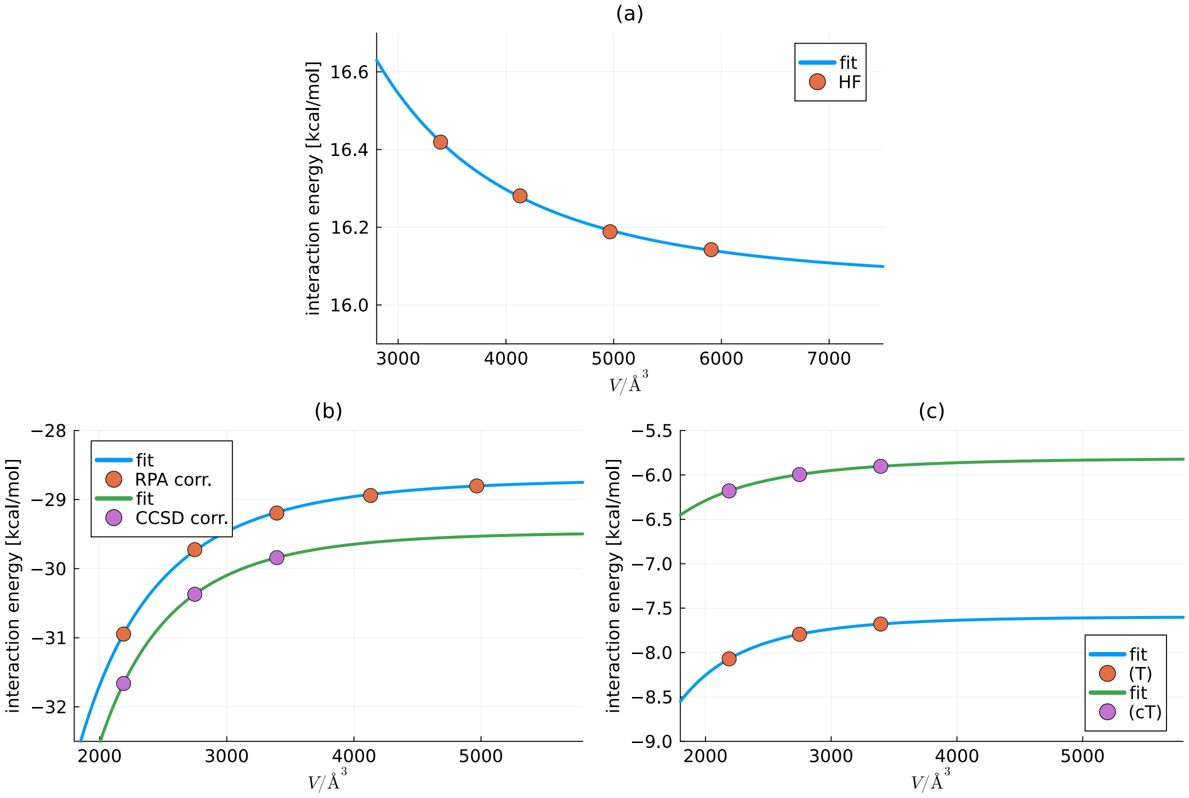

Fig. S 2. Box size dependence of the interaction energy of the coronene dimer for (a) HF, (b) CCSD, and the (c) triples. The CCSD and triples contribution was calculated with a basis set of $N_v/N_o = 15$ and $N_v/N_o = 12$, respectively.

The basis set dependence of the interaction energy is shown in Fig. S2. The additional focal point correction [10] dramatically reduces the basis set error of the CCSD energy and allows us to consider $N_v/N_o = 15$ as a very good approximation to the complete basis set limit. The triples contributions (T) and (cT) are corrected by rescaling the finite basis set result with a factor estimated on the level of MP2 theory as outlined in Ref [15]. Final CBS estimates of interaction energies for three systems in the L7 test set are provided in Table S III.

The timing for a plane wave based CCSD(cT) calculation of the coronene dimer system with the largest volume and $N_v/N_o = 10$ resulted in 100.4k CPU hours for the `Cc4s` program (step 6 in the workflow on p. 6) and 47.8k CPU hours for the steps 1 to 5.

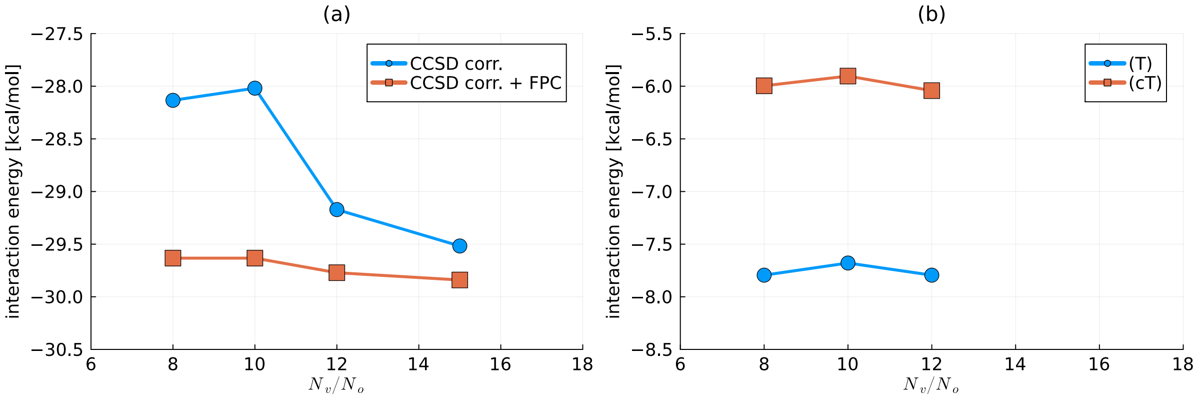

Fig. S 3. Basis set convergence of the correlation contribution of the interaction energy of the coronene dimer at the level of (a) CCSD and the (b) triples. A box size of 3375 $Å^3$ was considered. FPC denotes a basis set correction scheme cited in the text.

Table S III. Results of interaction energies in kcal/mol for three molecules from the L7 test set. CBS estimates obtained using the plane wave workflow as described in the text. LNO-CCSD(T) from GTO from literature are provided for comparison.

System	HF	MP2 corr.	CCSD corr.	(T)	(cT)	CCSD(T)	LNO-CCSD(T) [16]
GGG	7.725	-12.245	-7.771	-1.384	-1.130	-1.430	-2.1 ± 0.2
GCGC	12.407	-31.847	-21.193	-4.035	-3.343	-12.821	-13.6 ± 0.4
C2C2PD	16.096	-54.561	-29.471	-7.702	-5.949	-21.077	-20.6 ± 0.6

S5. ESTIMATING CCSD(CT)-FIT AND ITS UNCERTAINTY

As presented in Fig. 3 in the article, a linear trend can be observed, when plotting the ratio of (T) and (cT) against the ratio of the MP2 and CCSD correlation contribution of the interaction energies. The corresponding (T), (cT), MP2 as well as CCSD correlation energy contributions can be found in Table S IV.

A linear fit

$$\frac{\text{(T)}}{\text{(cT)}} = a + b \cdot \frac{\text{MP2 corr.}}{\text{CCSD corr.}} , \tag{2}$$

gives $a = 0.7764$ and $b = 0.2780$ with a standard deviation of the residuals of $\sigma = 0.0097$. Using this linear relationship, we derive the estimate CCSD(cT)-fit for the interaction energy of large molecules via

$$\text{CCSD(cT)-fit} = \text{LNO-CCSD} + \frac{1}{X} \cdot \text{LNO-(T)} , \tag{3}$$

where $X = a + b \cdot Y$ and Y is the ratio of the MP2 and CCSD correlation contribution obtained from the LNO coupled cluster approach.

This procedure allows us to calculate CCSD(cT) estimates for the large molecules presented in the work of Al-Hamdani and Nagy *et al.* [16]. Therefore we calculate $\Delta = \text{CCSD(T)} - \text{CCSD(cT)}$ using the LNO-CCSD(T) results from Table S V together with Eq. 2. This energy difference Δ is subtracted from the the well-converged LNO-CCSD(T) estimates provided in Ref. [16].

We estimate the uncertainty of the CCSD(cT)-fit interaction energy as the sum of the LNO-CCSD(T) uncertainty provided in Ref. [16] and an uncertainty from the fit. The former is a consequence of the tightness parameters controlling the local approximation. The latter can simply be calculated via the standard deviation of the residuals σ, which provides an error estimate for X. In correspondence with the uncertainty of the DMC results, which take 2σ, our corresponding error estimate for $1/X$ is thus given by $2\sigma/X^2$. Hence, the uncertainty measure for the fit depends on the considered molecular system but roughly takes the value of $2\sigma/X^2 \approx 0.025$ for all considered cases. Finally. this leads to an error estimate of

$$\delta\big(\text{CCSD(cT)-fit}\big) = \delta\big(\text{LNO-CCSD(T)}\big) + \left| \frac{2\sigma}{X^2} \cdot \text{LNO-(T)} \right| . \tag{4}$$

In fact, we make the simplification that $\delta\big(\text{LNO-CCSD(T)}\big)$ and $\delta\big(\text{LNO-CCSD}\big)$ are similar, as only the former is provided in Ref. [16].

Table S IV. Interaction energies in kcal/mol of a set of dispersion-dominated complexes from the S22, L7 and S66 benchmark datasets. Systems from the S22 test set are taken from Table S II and are calculated using cc-pVDZ basis sets. Systems from the S66 are obtained from [34] extrapolation and were taken from Table S I. Results for the three molecules from the L7 test set are obtained from plane wave calculations and were taken from Table S III.

System	MP2 corr.	CCSD corr.	(T)	(cT)
Methane dimer	-0.619	-0.559	-0.064	-0.059
Ethene dimer	-1.797	-1.368	-0.244	-0.218
Benzene-Methane complex	-2.424	-1.810	-0.299	-0.260
Benzene dimer PD	-8.312	-5.361	-1.025	-0.849
Pyrazine dimer	-9.111	-5.685	-1.126	-0.930
Indolebenzene complex stack	-12.640	-8.028	-1.587	-1.308
Pyridine-Pyridine PD	-9.339	-5.794	-1.310	-1.080
Pyridine-Pyridine TS	-5.258	-3.597	-0.762	-0.642
Benzene-Pyridine PD	-9.052	-5.651	-1.282	-1.058
Benzene-Pyridine TS	-5.119	-3.488	-0.735	-0.618
Pyridine-Uracil PD	-10.701	-7.110	-1.637	-1.362
Benzene-Benzene PD	-8.665	-5.423	-1.234	-1.019
Benzene-Benzene TS	-5.194	-3.518	-0.748	-0.628
Uracil-Uracil PD	-11.525	-8.142	-1.972	-1.669
Benzene-Uracil PD	-10.982	-7.334	-1.670	-1.394
GGG	-12.245	-7.771	-1.384	-1.130
GCGC	-31.847	-21.193	-4.035	-3.343
C2C2PD	-54.561	-29.471	-7.702	-5.949

Table S V: Results for the L7 molecules and C_{60}[6]CPPA using the LNO-CCSD(T) algorithm in `MRCC`. We use aug-cc-pVTZ basis sets and employ counterpoise correction. In all calculations we use as LNO threshold the keyword Tight. CCSD and (T) correlation energy contain each half of the MP2 correction originating from weak pairs. In addition to the individual energy contributions we show our LNO-CCSD(T) results in comparison with the CBS estimates published in Ref. [16]. Δ is the estimated difference between (T) and (cT) based on the described fitting procedure.

System	HF	MP2 corr.	CCSD corr.	(T)	LNO-CCSD(T)	LNO-CCSD(T) [16]	Δ
GGG	8.160	-12.365	-8.380	-2.142	-2.362	-2.100	-0.337
GCGC	12.317	-30.221	-20.840	-5.305	-13.828	-13.600	-0.808
C2C2PD	15.974	-53.177	-29.888	-8.091	-22.005	-20.600	-1.725
C3A	9.291	-35.507	-20.709	-5.848	-17.266	-16.500	-1.181
PHE	-13.644	-11.463	-8.109	-2.641	-24.394	-25.400	-0.383
C3GC	17.486	-61.393	-36.794	-10.305	-29.614	-28.700	-1.996
C_{60}@[6]CPPA	56.349	-142.987[17]	-78.723[17]	-21.907[17]	-44.280[17]	-41.700	-4.81[17]

S6. JUSTIFICATION OF CCSD(CT)-FIT

To better justify the linear fit defined by Eq. 2 and used for Fig. 3 in the article, we investigate additional systems, where the observed linear trend between the MP2/CCSD and (T)/(cT) ratios can be more clearly rationalized. These systems can be divided into two groups. Group I consists of parallel displaced polycyclic aromatic hydrocarbons (Benzene, Pyrene, Coronene), whereas group II consists of a single adenine molecule on top of a polycyclic aromatic hydrocarbon (Benzene, Naphthalene, Pyrene, Coronene). Their structures are depicted in Fig. S4. Further details on the employed geometries are provided in Section section S9. Furthermore, Fig. S4 illustrates that both groups yield (T)/(cT) ratios that are linearly dependent on the respective MP2/CCSD ratios. Note that MP2, CCSD, (T) and (cT) refer to correlation energy contributions to interaction energies. As in Fig. 3 of the article, these systems are dispersion-dominated complexes.

Our choice of systems is partly motivated by other studies, which focused on the calculation of interaction energies between a single water molecule and a monolayer of graphene or h-BN, see Refs. [18–20]. In particular, Refs. [18–20] have shown that the interaction energies between water and increasingly large planar molecules used to model the substrate converge in a systematic manner. This motivates extrapolation techniques to estimate the interaction energy in the limit $N \to \infty$, where N corresponds to the number of atoms in the substrate model. In the case of h-BN, it was observed that the interaction energy for large N can be well approximated by

$$E_{\text{int},X}(N) \approx E_{\text{int},X}(\infty) + \frac{A_X}{N^2}.$$

The $\frac{A_X}{N^2}$ term is motivated by pairwise additive dispersion interaction energy contributions. A_X and $E_{\text{int},X}(\infty)$ depend on the level of theory denoted as X. In the limit $N \to \infty$, it follows that the ratio between the interaction energies of two different

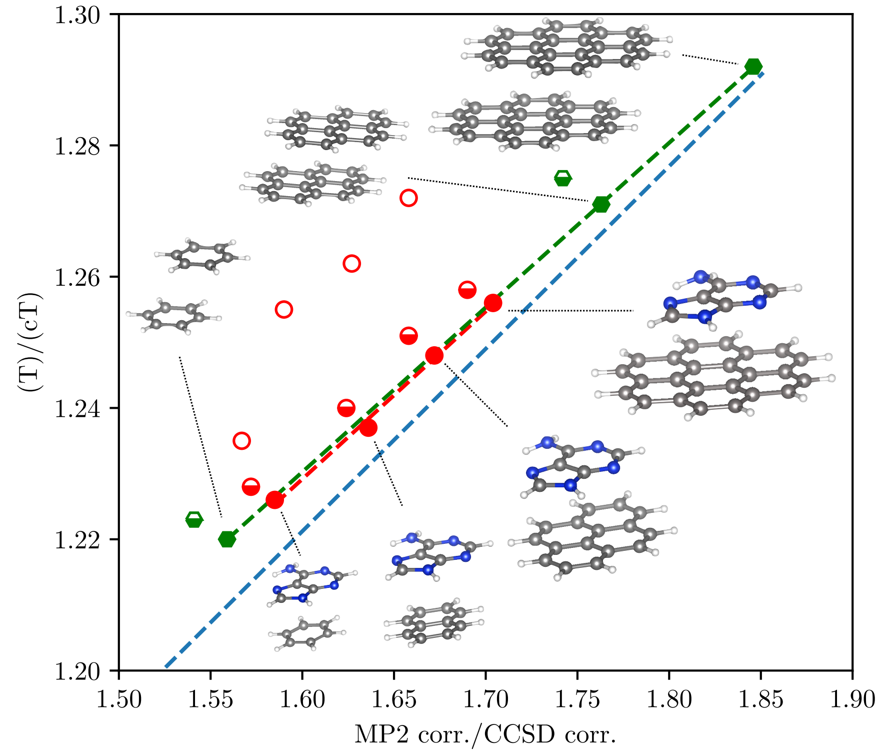

Fig. S 4. (T)/(cT) ratios retrieved as a function of MP2/CCSD ratios. Here, MP2, CCSD, (T) and (cT) stand for correlation energy contributions to interaction energies of a set of dispersion-dominated complexes shown. Empty, half and full symbols correspond to calculations employing N_v/N_o= 8, 10 and 12, respectively. The dashed lines correspond to fits according to Eq. 2. Green/red dashed lines were fitted to filled green/red data points. The blue dashed line is taken from the fit shown in Fig. 3 in the article.

theories X and X' becomes

$$\frac{E_{\text{int},X}(N)}{E_{\text{int},X'}(N)} = \frac{E_{\text{int},X}(\infty)}{E_{\text{int},X'}(\infty)} + \frac{p}{N^2} + \mathcal{O}(1/N^4),$$

where $p = \frac{A_X E_{\text{int},X'}(\infty) - E_{\text{int},X}(\infty) A_{X'}}{E_{\text{int},X'}(\infty)^2}$. Consequently, the ratios of MP2/CCSD and (T)/(cT) also become linearly dependent for systems with large N. This is a formal justification of Eq. 2 for systems similar to group II in the limit of sufficiently large N. However, Fig. S4 demonstrates that already relatively small N suffice for an excellent linear relationship between the ratios of MP2/CCSD and (T)/(cT). Moreover, we note that Eq. 2 also works reliably for systems from group I and more diverse systems studied in the article.

Note that Fig. S4 depicts linear fits of the ratios obtained for group I, II and the fit from the main article, which was performed for a more diverse set of systems. It can be seen that all fits are very similar and their ratios deviate at most by a few percent. Therefore, we argue that CCSD(cT)-fit is well suited for the systems studied in the present work. However, the reliability of CCSD(cT)-fit for systems that are very different from the polycyclic aromatic hydrocarbons investigated in this work cannot be assumed. This is already indicated by the small but significant difference in the slopes of group II and from the main article. Moreover, the system in the present work which has the least similarity to those in group I and II is C60@[6]CPPA, because it lacks a planar geometry. Nevertheless, it is currently not possible to obtain a more precise (cT) estimate for C60@[6]CPPA due to the size of the system.

The ratios depicted in Fig. S4 were computed in the following manner. Empty, half and full symbols were computed using 8, 10 and 12 virtual natural orbitals per occupied orbital. The change from 10 to 12 virtual natural orbitals per occupied orbital is relatively small, indicating that the remaining BSIE is negligible. These calculations use aug-cc-pVTZ basis sets and employ counterpoise correction. The required intermediates were computed with `MRCC` interfaced to the MP2 and CC algorithms implemented in `Cc4s`. The structures of the molecules were optimized using `MRCC` with the TPSS functional and def2-tzvp basis sets. We include the employed structures in the SI as xyz-files.

S7. ESTIMATING CCSD(CT) FOR PHE AND C3A

The CCSD(cT) interaction energies for PHE and C3A in Table 2 of the article were estimated using the LNO-CCSD(T) result and adding the explicitly computed (cT)-(T) estimate, which converges rapidly with respect to the basis set size.

For PHE, we obtain a (cT)-(T) contribution to the interaction energy of 0.25 kcal/mol and 0.28 kcal/mol using 8 and 10 virtual natural orbitals per occupied orbital, respectively. Although the employed basis is too small for well converged (T) interaction

energies, we find that the difference between (cT) and (T) is already well converged. This is also confirmed by looking at the difference between MP2 and CCSD correlation energy contributions to the interaction energies. The difference between CCSD and MP2 interaction energies using 8 and 10 virtual natural orbitals per occupied is 2.53 kcal/mol and 2.97 kcal/mol, respectively. This is close to the corresponding LNO estimate from Tab.SV, which is 3.35 kcal/mol.

In the case of C3A, we obtain a (cT)-(T) contribution to the binding energy of 0.73 kcal/mol and 0.47 kcal/mol using 6 and 10 virtual natural orbitals per occupied, respectively. Again the corresponding CCSD-MP2 estimates using 6 and 10 virtual natural orbitals per occupied orbital are 14.14 kcal/mol and 14.88 kcal/mol, comparing well to the 14.80 kcal/mol estimate from Tab.SV.

The individual MP2, CCSD and CCSD(T) correlation energy contributions to the interaction energies are not sufficiently well converged when using 10 virtual natural orbitals per occupied orbital, which is the reason why they are not reported. However, we emphasize again that the differences MP2-CCSD and (T)-(cT) are found to converge much faster.

The C3A and PHE calculations were performed using the `MRCC` interfaced to the MP2 and CC algorithms implemented in `Cc4s`. An aug-cc-pVQZ basis set and counterpoise corrections were employed.

S8. S22 DATA SET RESULTS FOR PERTURBATIVE TRIPLES METHODS

Here we perform a more detailed analysis of the triples correlation contribution for the S22 data set. Fig. S6 shows the comparison between the full triples energy E_T and a selection of perturbative triples methods for the set of molecules as listed in Table SII.

The perturbative triple theories that we compare are the (T), (cT), [T], and (cT)-ring methods. A discussion of the (cT) and (T) results is readily presented in the main manuscript. The results of the other two methods provide a better understanding of the different contributions appearing in the full CCSDT model. Fig. S6 illustrate the relevant perturbative triples diagrams. In this context, the (T) is represented by the energy contributions given by the S_1 and D_1 diagrams. The (cT) energy additionally contains contribution from all other presented diagrams. The full set of equations can be found in the Supplementary information of [21]. The [T] theory, also known as T(CCSD) [22], is given by solely considering D_1-like diagrams, i.e., leaving out the singles contributions represented by the S_1. This is in contrast to (T) and (cT) theories, which both incorporate these singles contributions. Our results show that excluding the S_1 diagram has little impact on the total energy quality compared to (T), and importantly, does not improve upon (T) for interaction energies. Similarly, excluding the corresponding singles contributions, S_1 - S_4, has only minor effect on the (cT) energies (not shown). We further investigate the different (cT) contributions by adding two particular diagrams to the (T) energy, namely diagrams D_2 and S_2. Results from this approach are denoted as (cT)-ring in the following. Figure S5(b) clearly demostrates that this contribution improves the agreement of interaction energies between CCSDT and the applied perturbative triples approach. It was argued early on that the inclusion of these particular terms is less justified if terms which couple the triples amplitudes with each other are disregarded [23]. These arguments might help to understand why the total energies of (cT) and (cT)-ring are systematically underestimated.

We stress that these contributions are nonetheless required to avert the systematic overestimation of interaction energies in the (T) model and there is no reason to expect that they can be cancelled consistently by higher terms in the cluster expansion.

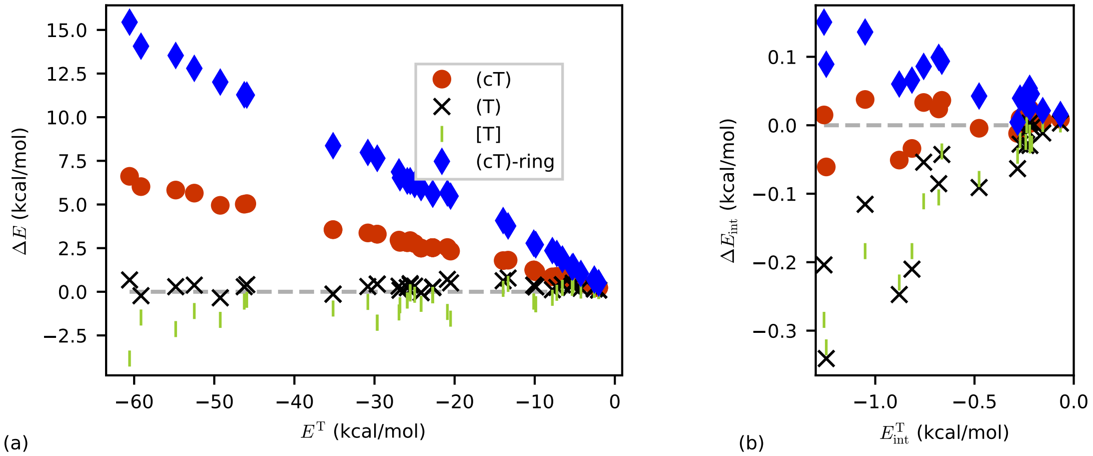

Fig. S 5. Comparison between the full triples and the perturbative triples approaches for a set of molecules contained in the S22 data set [24]. The total triples correlation energy contribution E^{T} on the x-axis is compared to both differences between the (T), (cT), [cT], [T] and (cT)-R correlation energy contributions and E^{T} for **a)** total energies and **b)** interaction energies.

S9. OVERVIEW OF EMPLOYED AND NEWLY GENERATED STRUCTURES

Most of the employed structures are taken from the S22, S66, and L7 benchmark sets. Beyond that, we introduce a new class of structures in section S6. In what we define as group 1, we worked with parallel-displaced polycyclic aromatic hydrocarbons. The

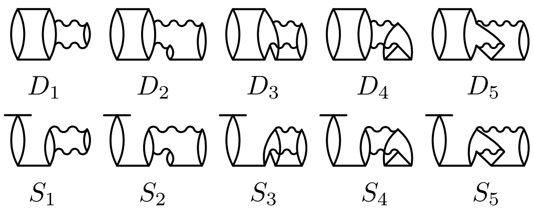

Fig. S 6. A selection of Goldstone diagrams contributing to the perturbative triples correction for a variety of perturbative theories. For the sake of conciseness, we do not write all permutations of the diagrams needed to evaluate the energy.

parallel-displaced benzene dimer is taken from the S66 benchmark set, and the coronene dimer is taken from the L7 benchmark set – these structures are similar to those used throughout the rest of the study. The pyrene dimer was relaxed using `NWCHEM` [9] with the TPSS functional combined with DFT-D3 dispersion correction and the def2-TZVP basis set. For group 2, comprising adenine adsorbed on a polycyclic aromatic hydrocarbon, we used the same optimization strategy. The coordinates of the newly generated structures are provided below.

Pyrene dimer

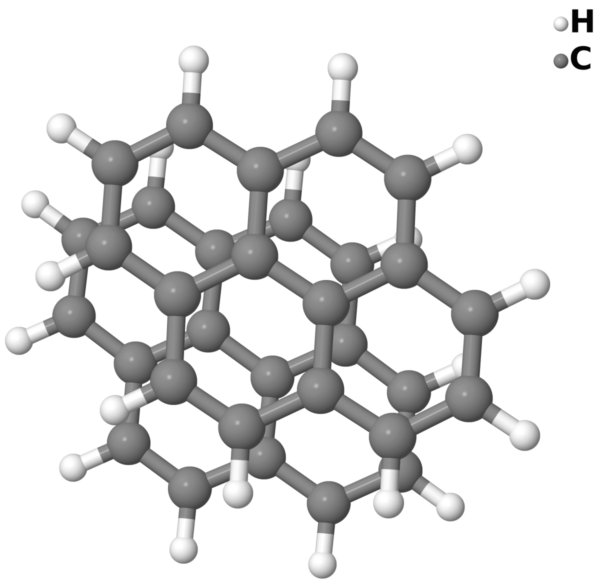

52

C	2.55711313	-1.30493740	-1.39232860
C	2.40232776	-2.68814536	-1.32704223
C	1.13786069	-3.26634486	-1.41366460
C	-1.32658033	-3.02329468	-1.65883289
C	-0.00759071	-2.46761425	-1.57381243
C	1.44317805	-0.46503188	-1.55236172
C	0.14266411	-1.04894160	-1.64870995
C	-1.00129719	-0.21781092	-1.81215081
C	-2.30319620	-0.80015495	-1.89809651
C	-2.42351665	-2.22665852	-1.81171835
C	-1.99303686	1.99973238	-2.05315149
C	-0.84983226	1.20116746	-1.88548500
C	0.46686434	1.75864973	-1.77182314
C	1.56216502	0.96286863	-1.61195690
C	-3.41682543	0.04133151	-2.06318454
C	-3.25886630	1.42347524	-2.14366930
H	3.54509379	-0.86082420	-1.30189915
H	3.27619967	-3.32067654	-1.19478975
H	-4.13181224	2.05895963	-2.26951761
H	-1.88027619	3.08023639	-2.09911263
H	2.55140324	1.40170067	-1.50802062
H	0.57311765	2.84046814	-1.80002728
H	-4.40893279	-0.40014457	-2.12950074
H	-3.41700032	-2.66588633	-1.87461432
H	-1.43669448	-4.10392375	-1.59766455
H	1.02601654	-4.34676529	-1.35567433
C	3.44555458	0.07784440	2.02176563
C	3.34266779	-1.30888294	2.11290805
C	2.09897483	-1.93410488	2.04815050
C	-0.37185822	-1.79106603	1.80513137
C	0.92315342	-1.18189030	1.89451696
C	2.29741807	0.87467660	1.87135756
C	1.01786896	0.24127367	1.80968621
C	-0.15981534	1.02581324	1.65606169
C	-1.43737720	0.39039882	1.57924947
C	-1.49956439	-1.04027747	1.65390911
C	-1.24350742	3.20090655	1.41682671
C	-0.06621540	2.44888737	1.57003991
C	1.23108470	3.05645539	1.63472401
C	2.36049580	2.30444208	1.77631754
C	-2.58500977	1.18515953	1.42351157
C	-2.48516110	2.57263419	1.34556772
H	4.42075773	0.55784304	2.06911065
H	4.24159103	-1.90925206	2.22724413
H	-3.38422554	3.16945965	1.21576017
H	-1.17489430	4.28458365	1.34939171
H	3.33668292	2.78294200	1.82150809
H	1.29768793	4.14033240	1.56590233
H	-3.55603123	0.70235184	1.34793750
H	-2.47183296	-1.51934964	1.56978700
H	-0.43503357	-2.87574142	1.84477625
H	2.02908007	-3.01766687	2.10190783

Adenine on Benzene

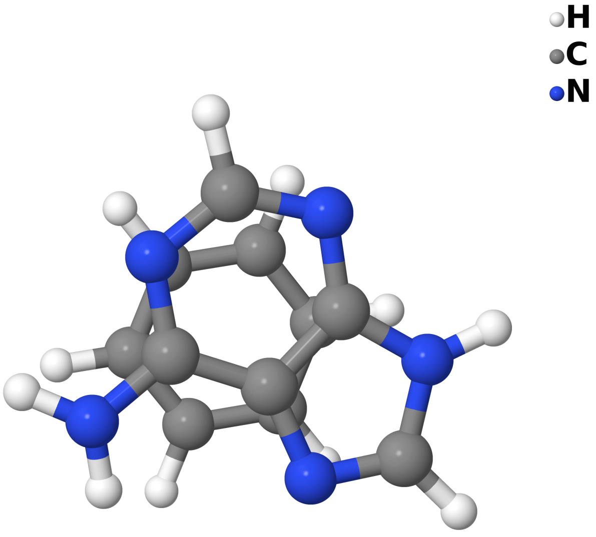

27

C	-1.08938670	-0.15978942	1.50100022
C	0.23050761	-0.58000046	1.24537678
C	1.19478427	0.43401077	1.18841889
C	-0.29422195	2.00753001	1.60619591
C	2.06482103	-1.56937033	0.81196991
N	-1.32928124	1.15276636	1.68062721
N	0.99786189	1.74766053	1.36253828
N	0.79104173	-1.82686080	1.00859380
N	2.37295139	-0.22620451	0.90902637
N	-2.12740661	-1.03241652	1.60439388
H	-0.54608937	3.05474416	1.75984924
H	2.82475516	-2.30848348	0.59455457
H	3.27984946	0.20577138	0.79310551
H	-3.05999221	-0.64268161	1.58553000
H	-1.99613396	-1.97074291	1.25250447
C	0.93336808	0.27598556	-2.32058298
C	0.41138146	-1.00760181	-2.48636328
C	-0.95457637	-1.23329719	-2.30635785
C	-1.79653651	-0.17497068	-1.96135112
C	0.09225357	1.33388050	-1.96920275
C	-1.27306440	1.10826758	-1.78920104
H	1.99771575	0.45172340	-2.45531419
H	1.06777721	-1.83370096	-2.74677108
H	-1.35998291	-2.23395122	-2.43213710
H	-2.86082654	-0.34905147	-1.82287813
H	0.50305374	2.32784855	-1.81769067
H	-1.92575590	1.92728060	-1.50114297

Adenine on Naphthalene

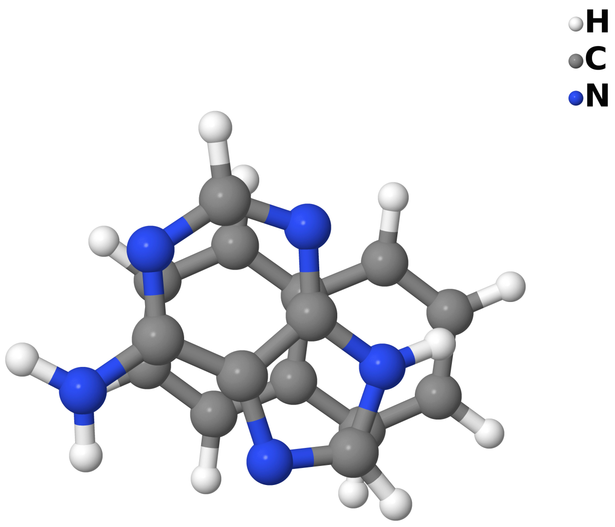

33

C	-1.73053413	0.01436241	2.06609213
C	-0.47924829	-0.54141747	1.73710774
C	0.56906165	0.37027326	1.55965940
C	-0.73131161	2.09695295	2.01193917
C	1.20844163	-1.71723203	1.19221782
N	-1.83410493	1.35068394	2.19995598
N	0.50787483	1.70361189	1.68739043
N	-0.06196908	-1.84512368	1.50857624
N	1.65240588	-0.41138232	1.21929624
N	-2.83582299	-0.74929824	2.26920147
H	-0.87165763	3.16898195	2.13460236
H	1.87074229	-2.52987200	0.92728561
H	2.54577663	-0.06848761	0.89020041
H	-3.72849479	-0.28283803	2.34306267
H	-2.81079722	-1.72284796	2.00335353
C	1.45406040	-1.50995241	-2.17582636
C	2.71658269	-0.95893279	-2.21933870
C	2.89628823	0.42994053	-2.00429044
C	1.80935894	1.24142013	-1.75716758
C	0.49578999	0.70430199	-1.70864404
C	0.31436176	-0.70327983	-1.91595100
C	-0.99424236	-1.24606862	-1.83143693
C	-2.07404656	-0.43733828	-1.55555058
C	-0.63953418	1.50991053	-1.43265238
C	-1.89580193	0.95214338	-1.35469048
H	-1.12725666	-2.31612097	-1.97081127
H	-0.49585911	2.57264995	-1.25779767
H	1.31307828	-2.57680294	-2.33485988
H	1.94388124	2.30580544	-1.58146076
H	-3.06972943	-0.86612387	-1.47898566
H	-2.75330441	1.57541942	-1.11827394
H	3.57979322	-1.58786982	-2.42154088
H	3.89616127	0.85494145	-2.04207137

Adenine on Pyrene

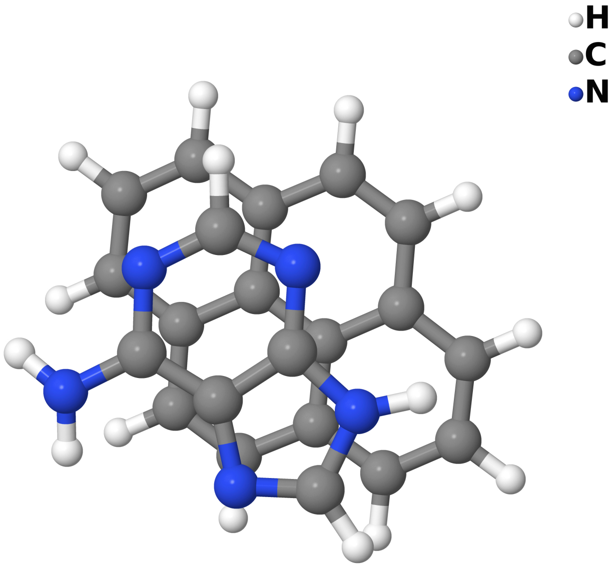

41

C	-1.95197867	-0.27259292	1.81562118
C	-0.75243142	-1.00248906	1.91598358
C	0.41198815	-0.24316602	2.09684535
C	-0.71341461	1.65595361	2.10814378
C	0.84579332	-2.40910915	1.93712941
N	-1.90746698	1.06885854	1.91712572
N	0.49692417	1.08859847	2.20925940
N	-0.46308114	-2.35759314	1.82321010
N	1.43083064	-1.17210473	2.11744345
N	-3.16377767	-0.87356916	1.64559602
H	-0.73619656	2.74092361	2.17983227
H	1.44053269	-3.31142670	1.89669187
H	2.42012461	-0.96196504	2.12425992
H	-3.92256987	-0.27616018	1.34537728
H	-3.16886324	-1.83259978	1.32595829
C	3.36640610	-0.39814882	-0.86795908
C	3.22053738	-1.77832927	-1.00951641
C	1.96935618	-2.33734001	-1.26514769
C	-0.47542196	-2.06230033	-1.63178478
C	0.82958601	-1.52312896	-1.38368099
C	2.25717559	0.45819941	-0.98338614
C	0.97032579	-0.10799387	-1.24124902
C	-0.17102861	0.73584669	-1.34664819
C	-1.45932229	0.16985002	-1.59149270
C	-1.56806615	-1.25276677	-1.73256545
C	-1.17597664	2.95916009	-1.28504809
C	-0.03124430	2.14961473	-1.19790229
C	1.27415886	2.68902434	-0.95250124
C	2.36716371	1.88147753	-0.84782555
C	-2.57317211	1.02195820	-1.67355385
C	-2.43002378	2.39860295	-1.51749212
H	4.34782674	0.03059336	-0.67676789
H	4.09187152	-2.42323319	-0.92567246
H	-3.30459052	3.04101891	-1.57295864
H	-1.07382294	4.03476779	-1.16144248
H	3.34790298	2.30761525	-0.64938038
H	1.37393156	3.76573417	-0.83616152
H	-3.55568465	0.59217991	-1.85616739
H	-2.55158973	-1.67908091	-1.91900315
H	-0.57905699	-3.14044575	-1.72558928
H	1.86299000	-3.41432417	-1.37261215

Adenine on Coronene

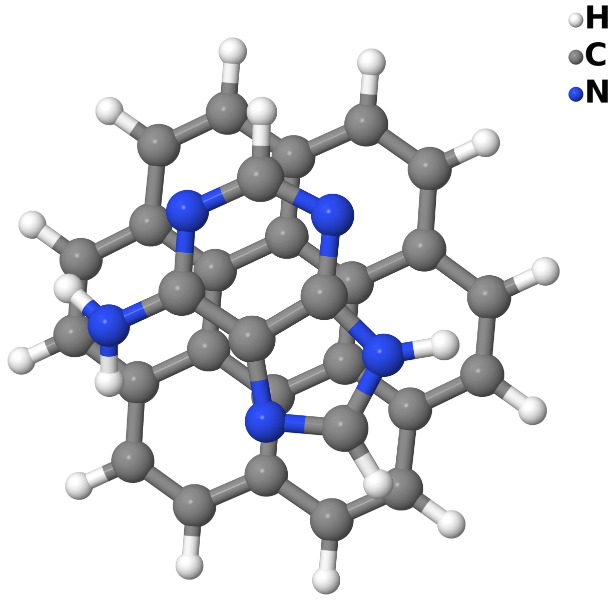

51

C	-1.53776385	0.35848106	2.14891608
C	-0.37055136	-0.41914982	2.27695252
C	0.82163172	0.29048833	2.47543485
C	-0.22617114	2.23364829	2.47597018
C	1.16738297	-1.89248398	2.30773547
N	-1.43992313	1.69722464	2.26083318
N	0.95956493	1.61828978	2.59087946
N	-0.13622292	-1.78415970	2.17758282
N	1.80105700	-0.68035105	2.50112075
N	-2.76418484	-0.19456672	1.93010044
H	-0.20593176	3.31845554	2.55297095
H	1.72277594	-2.81908170	2.26308056
H	2.79780893	-0.51203555	2.53303009
H	-3.46982640	0.42883334	1.55865582
H	-2.77694779	-1.14399943	1.57964963
C	3.78007311	0.29501042	-0.48340034
C	3.66561633	-1.07178343	-0.58217827
C	2.40520811	-1.69132212	-0.81113712
C	2.25226990	-3.10331559	-0.89768798
C	1.01800217	-3.67528118	-1.09116351
C	-0.15248794	-2.87745905	-1.21680145
C	-0.02840356	-1.46198473	-1.14556350
C	1.25103891	-0.86778563	-0.94054260
C	2.64465580	1.14265027	-0.61322895
C	1.37161606	0.54951518	-0.84430661
C	0.21425392	1.37299733	-0.96261466
C	-1.06489186	0.77912016	-1.16798743
C	-1.18649920	-0.63839700	-1.25772701
C	-1.44339624	-3.44418047	-1.40228939
C	-2.56013992	-2.65137653	-1.51156785
C	-2.46693730	-1.23312031	-1.44212436
C	-3.60708076	-0.38671740	-1.54138696
C	-2.22287304	1.60105751	-1.26279368
C	-3.48885457	0.98010010	-1.45382482
C	-2.07367681	3.01074195	-1.15299147
C	-0.84050776	3.58252739	-0.95937615
C	0.33370836	2.78672967	-0.85663681
C	1.61991751	3.35123550	-0.63457045
C	2.73395695	2.55825541	-0.51349186
H	4.75264084	0.75056035	-0.30939059
H	4.54747062	-1.70230777	-0.48766300
H	3.13630648	-3.73054559	-0.80268473
H	0.92005357	-4.75709850	-1.14717410
H	-1.53532008	-4.52673561	-1.45306581
H	-3.53943759	-3.10356732	-1.65337414
H	-4.58396120	-0.84264544	-1.68846988
H	-4.37205492	1.61070544	-1.53055436
H	-2.96151915	3.63549390	-1.21712735
H	-0.74731296	4.66250898	-0.87066536
H	3.70717498	3.00779228	-0.33054273
H	1.70678154	4.43183629	-0.54793830

[1] M. Kállay, P. R. Nagy, D. Mester, Z. Rolik, G. Samu, J. Csontos, J. Csóka, P. B. Szabó, L. Gyevi-Nagy, B. Hégely, I. Ladjánszki, L. Szegedy, B. Ladóczki, K. Petrov, M. Farkas, P. D. Mezei, and A. Ganyecz, The Journal of Chemical Physics **152**, 074107 (2020).

[2] P. R. Nagy and M. Kállay, J. Chem. Phys. **146**, 214106 (2017).

[3] P. R. Nagy, G. Samu, and M. Kállay, J. Chem. Theory Comput. **14**, 4193 (2018).

[4] P. R. Nagy and M. Kállay, J. Chem. Theory Comput. **15**, 5275 (2019).

[5] `CC4S` developer team, "`CC4S` user manual," `https://manuals.cc4s.org/user-manual/` (2024).

[6] B. D. L. Péter R. Nagy, László Gyevi-Nagy and M. Kállay, Molecular Physics **121**, e2109526 (2023).

[7] Q. Ma and H.-J. Werner, Journal of Chemical Theory and Computation **15**, 1044 (2019).

[8] S. N. Kesharwani Manoj K., Karton Amir and M. J. M. L., Australian Journal of Chemistry **71**, 238 (2018).

[9] M. Valiev, E. Bylaska, N. Govind, K. Kowalski, T. Straatsma, H. Van Dam, D. Wang, J. Nieplocha, E. Apra, T. Windus, and W. de Jong, Comput. Phys. Commun. **181**, 1477 (2010).

[10] A. Irmler, A. Gallo, and A. Grüneis, The Journal of Chemical Physics **154**, 234103 (2021), 2103.06788.

[11] G. Kresse and J. Furthmüller, Computational Materials Science **6**, 15 (1996).

[12] T. Gruber, K. Liao, T. Tsatsoulis, F. Hummel, and A. Grüneis, Phys. Rev. X **8**, 021043 (2018).

[13] A. Grüneis, G. H. Booth, M. Marsman, J. Spencer, A. Alavi, and G. Kresse, J. Chem. Theory Comput. **7**, 2780 (2011).

[14] F. Hummel, T. Tsatsoulis, and A. Grüneis, The Journal of Chemical Physics **146**, 124105 (2017).

[15] G. Knizia, T. B. Adler, and H. J. Werner, Journal of Chemical Physics **130** (2009), 10.1063/1.3054300/908511.

[16] Y. S. Al-Hamdani, P. R. Nagy, A. Zen, D. Barton, M. Kállay, J. G. Brandenburg, and A. Tkatchenko, Nature Communications **12**, 3927 (2021).

[17] P. Nagy, personal communication.

[18] A. O. Ajala, V. Voora, N. Mardirossian, F. Furche, and F. Paesani, Journal of Chemical Theory and Computation **15**, 2359 (2019), pMID: 30860827.

[19] G. R. Jenness, O. Karalti, and K. D. Jordan, Phys. Chem. Chem. Phys. **12**, 6375 (2010).

[20] Y. S. Al-Hamdani, M. Rossi, D. Alfè, T. Tsatsoulis, B. Ramberger, J. G. Brandenburg, A. Zen, G. Kresse, A. Grüneis, A. Tkatchenko, and A. Michaelides, The Journal of Chemical Physics **147**, 044710 (2017).

[21] N. Masios, A. Irmler, T. Schäfer, and A. Grüneis, Phys. Rev. Lett. **131**, 186401 (2023).

[22] M. Urban, J. Noga, S. J. Cole, and R. J. Bartlett, The Journal of Chemical Physics **83**, 4041 (1985), https://pubs.aip.org/aip/jcp/article-pdf/83/8/4041/18955437/4041_1_online.pdf.

[23] Y. S. Lee, S. A. Kucharski, and R. J. Bartlett, The Journal of Chemical Physics **81**, 5906 (1984), https://pubs.aip.org/aip/jcp/article-pdf/81/12/5906/18952540/5906_1_online.pdf.

[24] P. Jurečka, J. Šponer, J. Černý, and P. Hobza, Phys. Chem. Chem. Phys. **8**, 1985 (2006).
