# Supplementary material for: Understanding discrepancies in noncovalent interaction energies from wavefunction theories for large molecules
Source: Nat Commun. 2025 Oct 14;16:9108. doi: 10.1038/s41467-025-64104-8 (PMC12521354; doi:10.1038/s41467-025-64104-8)
Supplement: Supplementary file 2 — Description of Additional Supplementary Files [file 41467_2025_64104_MOESM2_ESM.pdf]

Description of Additional Supplementary Files

File name: Supplementary Data 1
Description: xyz-files for additional structures that are not part of widely-used benchmark sets.
