# Supplementary material for: Understanding discrepancies in noncovalent interaction energies from wavefunction theories for large molecules
Source: Nat Commun. 2025 Oct 14;16:9108. doi: 10.1038/s41467-025-64104-8 (PMC12521354; doi:10.1038/s41467-025-64104-8)
Supplement: Supplementary file 4 — Transparent Peer Review file [file 41467_2025_64104_MOESM4_ESM.pdf]

nature portfolio

Peer Review File

Understanding Discrepancies of Wavefunction Theories for Large Molecules

Corresponding Author: Professor Andreas Grüneis

This file contains all reviewer reports in order by version, followed by all author rebuttals in order by version.

Version 0:

Reviewer comments:

Reviewer #1

(Remarks to the Author)
The manuscript explores the accuracy of a recently proposed approximation to the electronic correlation energy for large molecules.
There exists a bias in the electronic correlation community towards small molecular systems for which very accurate calculations are accessible.
The common wisdom and approximation design are over-weighting the small systems for sure.
It is therefore very interesting that researchers address larger systems today.

Here the authors focus in particular on the interaction energy of non-covalently-bound molecules.
The main argument of the authors is the following:
While their new approximation, named CCSD(cT), is worse than the usual perturbative CCSD(T) for correlation energies, it is a more robust approximation to full CCSDT for interaction energies.
It is correct that the physical and chemical properties are sensitive to interaction energies and not to the total energy.

However, I find that the data reported in the manuscript to support the claims are not compelling enough.
The improvement brought by CCSD(cT) over CCSD(T) is clear for the coronene dimer (C2C2PD) (~2 kcal/mol), but it is not very striking for all the other systems reported in figures 2 and 3 (~0.2 kcal/mol).
0.2 kcal/mol is way below the usually targeted "chemical accuracy".

The authors do a great job to obtain rock-solid values by using systematic basis, such as plane-waves, natural orbitals, system-size extrapolation.
Unfortunately, in the end (Table 2), they use a very lightly justified fit to estimate the CCSD(cT) results based on extrapolating values obtained from a previous work.

More convincing data are necessary, in my opinion, to better support the authors' claim.

Minor remarks:

- Figure 1: y-label should be Delta E^int and not Delta E

- Sentence "CCSD(T) is often fortuitously closer to CCSDTQ than CCSDT.34"
If it is really so, CCSD(cT) should be compared to CCSDTQ not to CCSDT in Figure 2 for instance.

- Sentence "A similar problem is known to occur in MP2 theory"
The authors meant Moller-Plesset theory, not MP2.

Reviewer #2

(Remarks to the Author)

This manuscript reports interesting results indicating a possibility to treat noncovalent interactions with the coupled cluster (CC) approach.

The authors use the CCSD model (CC with full inclusion of singles and doubles) supplemented with the noniterative triples, altered with respect to the standard CCSD(T) model. The new scheme with the modified triple contribution was introduced in Ref. 30 and is named as CCSD(cT). The current work is aimed at the evaluation of the performance of the latter method when applied to the calculations of the energy of noncovalent interactions.

The studied examples involve the parallel displaced coronene dimer with respective results shown in Table 1. The wider range of noncovalently bound complexes includes a set of parallel displaced dimers built from the benzene, pyridine and uracil monomers. The computed interaction energies were compared to the reference values provided by DMC (Diffusion Monte Carlo) calculations, Fig. 1. The authors conclude that, both for the coronene dimer as well as for the set of smaller weakly bound complexes, the CCSD(cT) scheme provides significantly better, i.e. closer to the reference, results than the standard CCSD(T) scheme.

Another test was provided by the studies of the molecular examples collected in the S22 data set. Here the CCSD(T) and CCSD(cT) were compared to the full CCSDT results (Fig.2). It has been observed that the CCSD(cT) interaction energy of the noncovalently bound monomers is much closer to the full CCSDT value than the energy provided by the standard CCSD(T). However, the calculations of the total energies indicate a superiority of the regular CCSD(T) scheme (i.e. closer to the full CCSDT).

The results reported in the manuscript are worth of attention. However, I think that of more groundbreaking character is a work reported in Ref. 30 (mostly by the same authors) introducing the CCSD(cT) scheme. The current work provides the wider range of applications of the latter approach and turns our attention to use the CCSD(cT) scheme as a possible remedy for the deficiencies of a current gold standard scheme.

An unexpected consequence is that we face the situation when one approximate model works better for one molecular property (e.g., the CCSD(T) and the total molecular energy) while the other one (e.g. CCSD(cT)), apparently more elaborated, provides worse values for total energy but significantly better for the interaction energy. So we are loosing an important feature of the CC scheme, i.e. its systematic improvability.

The calculations reported in the manuscript are solid, done in careful manner to ensure sound conclusions. However, there is some inconsequence in the attached supplementary materials:
E.g., in Table SII there are columns headed by (T), (cT) and T, apparently providing the respective triple contributions. It seems that the (T) contribution is inconsistent with the values listed in other two columns. At the beginning of the section S6 there are some remarks concerning Fig. S2, but the text does not correspond to the content of Fig. S2.

In summary: I consider this work as valuable position in the studies of the coupled cluster theory.
I recommend the manuscript to be published in the Nature Communications after minor modifications.

Version 1:

Reviewer comments:

Reviewer #1

(Remarks to the Author)
I thank the authors for the additional data they included in the supplemental information.

I am still puzzled by the superiority of CCSD(T) over CCSD(cT) for total energies.
A lucky error cancellation that explains the success of CCSD(T) over the years.

Any way, the interaction energies for large molecules are worth being published.
I recommend publication as is.

Reviewer #2

(Remarks to the Author)
I am satisfied with the author's answers and I recommend the manuscript to be published in Nature Communications.

Open Access This Peer Review File is licensed under a Creative Commons Attribution 4.0 International License, which permits use, sharing, adaptation, distribution and reproduction in any medium or format, as long as you give appropriate credit to the original author(s) and the source, provide a link to the Creative Commons license, and indicate if changes were made.
In cases where reviewers are anonymous, credit should be given to 'Anonymous Referee' and the source.
The images or other third party material in this Peer Review File are included in the article's Creative Commons license, unless indicated otherwise in a credit line to the material. If material is not included in the article's Creative Commons license and your intended use is not permitted by statutory regulation or exceeds the permitted use, you will need to obtain permission directly from the copyright holder.
To view a copy of this license, visit https://creativecommons.org/licenses/by/4.0/

Below we address **report 1** in detail (reviewer's comments are printed in bold fonts).

The manuscript explores the accuracy of a recently proposed approximation to the electronic correlation energy for large molecules. There exists a bias in the electronic correlation community towards small molecular systems for which very accurate calculations are accessible. The common wisdom and approximation design are over-weighting the small systems for sure. It is therefore very interesting that researchers address larger systems today.

We thank the reviewer for their time and useful comments that lead to significant improvements of our manuscript.

Here the authors focus in particular on the interaction energy of non-covalently-bound molecules. The main argument of the authors is the following: While their new approximation, named CCSD(cT), is worse than the usual perturbative CCSD(T) for correlation energies, it is a more robust approximation to full CCSDT for interaction energies. It is correct that the physical and chemical properties are sensitive to interaction energies and not to the total energy.

We fully agree with the reviewer's remarks regarding total and interaction energies.

However, I find that the data reported in the manuscript to support the claims are not compelling enough. The improvement brought by CCSD(cT) over CCSD(T) is clear for the coronene dimer (C2C2PD) (~2 kcal/mol), but it is not very striking for all the other systems reported in figures 2 and 3 (~0.2 kcal/mol). 0.2 kcal/mol is way below the usually targeted "chemical accuracy".
The authors do a great job to obtain rock-solid values by using systematic basis, such as plane-waves, natural orbitals, system-size extrapolation. Unfortunately, in the end (Table 2), they use a very lightly justified fit to estimate the CCSD(cT) results based on extrapolating values obtained from a previous work. More convincing data are necessary, in my opinion, to better support the authors' claim.

We would like to thank the reviewer for their comments and apologise for the fact that the previous version of our manuscript did not provide a sufficiently robust justification for the CCSD(cT)-fit estimate. We address this weakness in the resubmitted version by:
(i) adding additional results on the level of CCSD(cT) for C3A and PHE in Table 2.
(ii) providing a detailed justification for the fit procedure in the SI, alongside more results for a set of systems that underpin our assumptions.

The additional results for C3A and PHE in Table 2 have been obtained by converging the (cT)-(T) interaction energy contribution separately using up to 10 natural orbitals per occupied orbital. We verify that these corrections are well converged with respect to basis set size and add them to the LNO-CCSD(T) estimates. Our findings confirm the previous CCSD(cT)-fit results for the studied systems. A detailed summary of the calculations can be found in the SI (see S7).

We sincerely thank the reviewer for pointing out that the fit procedure was not sufficiently well justified. This motivated us to add a detailed justification and additional results assessing CCSD(cT)-fit for several systems depicted below (and in S6 of the updated SI).

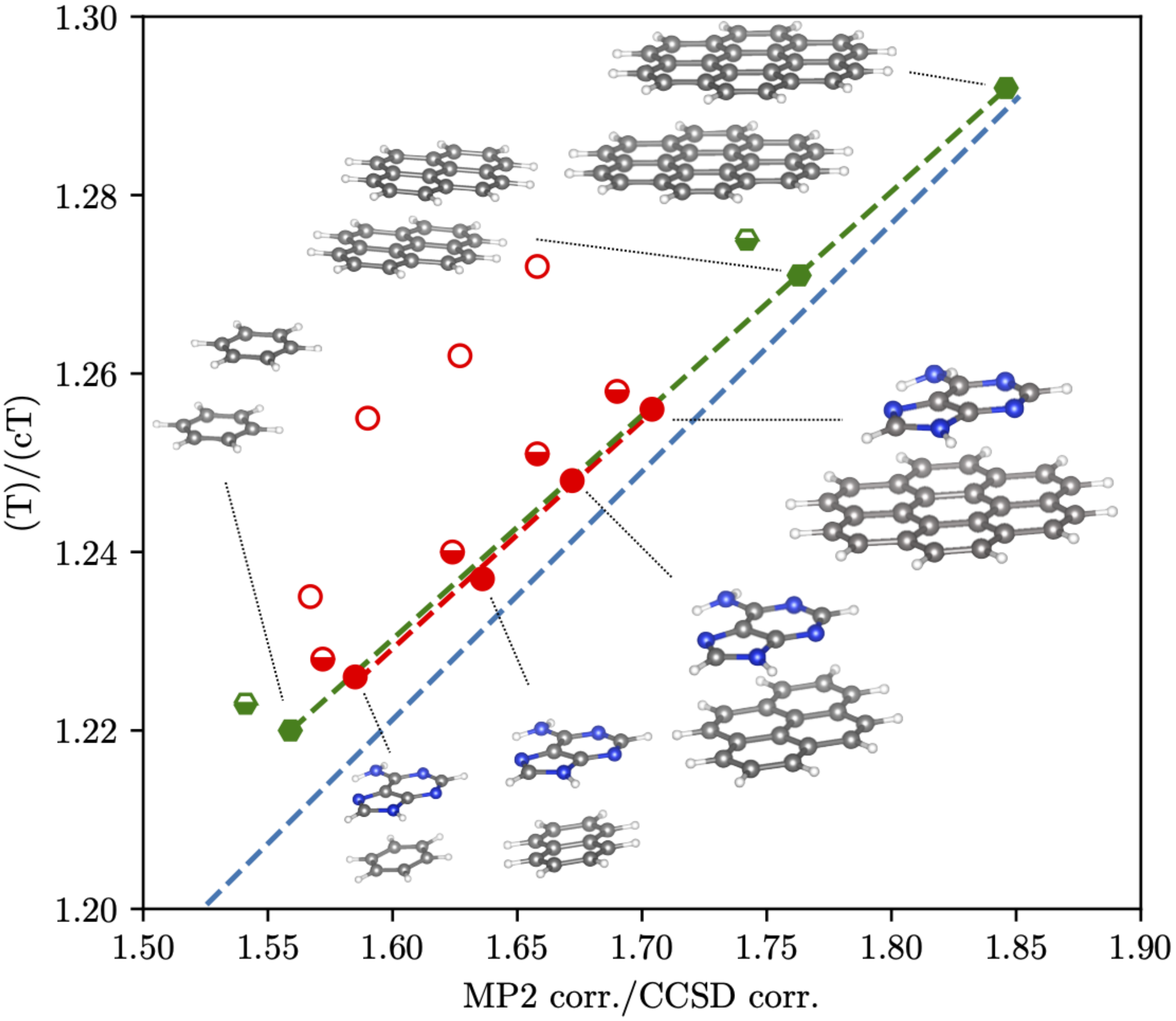

We justify CCSD(cT)-fit for the studied systems in more detail, by comparing to extrapolation methods used to estimate the interaction energy between molecules and substrates modelled by planar molecules. Assuming that different theories yield interaction energies that converge to the infinitely large substrate model limit with the same power law, we conclude that the employed linear relationship between the studied ratios becomes exact. However, we also point out that the CCSD(cT)-fit approach can be expected to work less reliable when fitting ratios of very different systems, as partly indicated by the small differences in the slopes of the systems studied in the article (blue dashed line) and the newly studied systems (depicted above). This difference in the ratios is, however, only a few percent at most and, therefore, is not expected to change the main conclusions of our work.

Minor remarks:
- Figure 1: y-label should be Delta E^int and not Delta E

We thank the reviewer for carefully reading our manuscript. This typo was corrected.

- Sentence "CCSD(T) is often fortuitously closer to CCSDTQ than CCSDT.34"
If it is really so, CCSD(cT) should be compared to CCSDTQ not to CCSDT in Figure 2 for instance.

In our opinion, the CCSD(T) and CCSD(cT) methods can only be meaningfully compared to the CCSDT theory. Although a better agreement between CCSD(T) and CCSDTQ theory exists in some cases, as has been found and discussed in the literature for small molecular systems, there is no reason to believe that this would be generally applicable. This is because these methods include completely different physical contributions.
As shown in this work there exists an algebraic contribution in (T) which systematically overestimates the interaction energy. Mathematically, this can only be corrected by the missing triples contributions as it is done in (cT). Therefore, any agreement between CCSD(T) and CCSDTQ would have to be fortuitous. Furthermore, we emphasize that CCSDTQ calculations are computationally even more demanding and currently not possible for such relatively large systems and basis sets.

- Sentence "A similar problem is known to occur in MP2 theory"
The authors meant Moller-Plesset theory, not MP2.

We thank the reviewer for pointing out this error, which was corrected.

Below we address **report 2** in detail (reviewer's comments are printed in bold fonts).

This manuscript reports interesting results indicating a possibility to treat noncovalent interactions with the coupled cluster (CC) approach. The authors use the CCSD model (CC with full inclusion of singles and doubles) supplemented with the noniterative triples, altered with respect to the standard CCSD(T) model. The new scheme with the modified triple contribution was introduced in Ref. 30 and is named as CCSD(cT). The current work is aimed at the evaluation of the performance of the latter method when applied to the calculations of the energy of noncovalent interactions.

We sincerely thank the reviewer for their time and useful comments that lead to further improvements of the manuscript.

The studied examples involve the parallel displaced coronene dimer with respective results shown in Table 1. The wider range of noncovalently bound complexes includes a set of parallel displaced dimers built from the benzene, pyridine and uracil monomers. The computed interaction energies were compared to the reference values provided by DMC (Diffusion Monte Carlo) calculations, Fig. 1. The authors conclude that, both for the coronene dimer as well as for the set of smaller weakly bound complexes, the CCSD(cT) scheme provides significantly better, i.e. closer to the reference, results than the standard CCSD(T) scheme. Another test was provided by the studies of the molecular examples collected in the S22 data set. Here the CCSD(T) and CCSD(cT) were compared to the full CCSDT results (Fig.2). It has been observed that the CCSD(cT) interaction energy of the noncovalently bound monomers is much closer to the full CCSDT value than the energy provided by the standard CCSD(T). However, the calculations of the total energies indicate a superiority of the regular CCSD(T) scheme (i.e. closer to the full CCSDT). The results reported in the manuscript are worth of attention. However, I think that of more groundbreaking character is a work reported in Ref. 30 (mostly by the same authors) introducing the CCSD(cT) scheme. The current work provides the wider range of applications of the latter approach and turns our attention to use the CCSD(cT) scheme as a possible remedy for the deficiencies of a current gold standard scheme. An unexpected consequence is that we face the situation when one approximate model works better for one molecular property (e.g., the CCSD(T) and the total molecular energy) while the other one (e.g. CCSD(cT)), apparently more elaborated, provides worse values for total energy but significantly better for the interaction energy. So we are loosing an important feature of the CC scheme, i.e. its systematic improvability. The calculations reported in the manuscript are solid, done in careful manner to ensure sound conclusions.
However, there is some inconsequence in the attached supplementary materials: E.g., in Table SII there are columns headed by (T), (cT) and T, apparently providing the respective triple contributions. It seems that the (T) contribution is inconsistent with the values listed in other two columns. At the beginning of the section S6 there are some remarks concerning Fig. S2, but the text does not correspond to the content of Fig. S2.

We are very grateful to the reviewer for carefully reading and understanding our manuscript.
We apologize for the formatting errors of Table SII and referencing errors at the beginning of section S6. These errors have been corrected in the resubmitted version.

In summary: I consider this work as valuable position in the studies of the coupled cluster theory. I recommend the manuscript to be published in the Nature Communications after minor modifications.
